# A statistical damage model for coupled mechano-electrophysiological axonal injury

**DOI:** 10.1371/journal.pcbi.1014837

**Published:** 2026-10-09

**Authors:** Zexuan Chen, Liqun Tang, Bao Yang, Yiping Liu, Zhenyu Jiang, Licheng Zhou, Zejia Liu

**Affiliations:** Department of Engineering Mechanics, School of Civil Engineering and Transportation, South China University of Technology, Guangzhou, Guangdong, China; University of Ottawa, CANADA

## Abstract

Deformation-induced injury of white matter nerve bundles is prevalent in central nervous system injuries, leading to a series of neurological dysfunctions. To describe the effect of deformation injury on the physiological electrical signals of neurons, several axonal electrophysiological damage models based on the Hodgkin-Huxley equations have been proposed. However, none of these models can fully characterize the results of white matter nerve stretching experiments under different rates and amplitudes. Building on previous work by peers, we propose a novel axonal deformation damage model that comprehensively considers the coupled effects of sodium and potassium channel damage on gating variable rate constants, as well as the differences in mechanical properties and injury among individual axons during deformation—with these differences continuously modeled by a statistical distribution. The proposed model can not only reasonably describe the sodium left-shift phenomenon (a key post-injury feature) but also accurately reproduce the neuro-electrophysiological responses of nerve fascicles under various stretching conditions. This new neuro-electrophysiological model for nerve fascicle injury exhibits good universality and provides a mechanical mechanism-based reference for future clinical treatment strategies of nerve injury.

## Author summary

Deformation-induced injury of white matter nerve bundles is a common pathology in traumatic central nervous system injuries. Existing models disagree on ion channel injury mechanisms and insufficiently match classic experimental data, requiring refinement. To address this, we modified existing models by innovatively integrating the comprehensive effects of sodium and potassium channel damage on gating rates, and introducing a statistical distribution for micro-axonal injury heterogeneity within nerve bundles. Results show that our model effectively compensates for the limitations of existing models. It not only provides a more unified and detailed explanatory framework for neural deformation injury but also lays a research foundation from a statistical perspective, promising to support traumatic brain injury clinical assessment and neuroprotective strategies development with mechanical basis.

## 1. Introduction

White matter nerve bundle injury is prevalent in traumatic central nervous system injuries and can cause various neurological dysfunctions. In traumatic brain injury (TBI), its mechanical injury manifests as diffuse axonal injury (DAI), which accounts for the second largest proportion of post-traumatic brain injury deaths [1,2], and is closely associated with neurodegenerative diseases, cognitive decline and other neuropsychiatric symptoms [3,4]; in traumatic spinal cord injury (TSI), white matter axonal damage caused by external forces leads to impairment or even loss of motor and sensory functions [5,6].

The core of white matter nerve bundle injury is microscopic axonal stretch injury: in traumatic brain injury (TBI), shear forces in brain tissue cause excessive stretching of neurons, damaging the axonal cytoskeleton and leading to axonal degeneration or even rupture [4,7]; in spinal cord mechanical injury, excessive longitudinal strain during lateral force application is the main form of compressive injury [8–10]. In vitro cellular injury experiments reveal that repeated stretching, even at mild magnitudes, induces severer cumulative damage in neurons and glia than single stretch loading. Morphologically, this manifests as disrupted cytoskeleton architecture and markedly elevated mechanical perforation of cell membranes, including growth cone collapse, co-localization of microtubules and actin, neuronal swelling, and release of intracellular specific biomarkers [11–14].

To thoroughly investigate the mechanical and physiological mechanisms underlying functional axonal stretch injury, researchers have developed numerous advanced computational and experimental models in recent years. For computational models, single-axon finite element models incorporating elaborate subcellular components including microtubules, neurofilaments, Tau proteins, axolemma and myelin sheath have been established. Uniaxial stretching simulations enable analysis of deformation and failure features of individual substructures, discrepancies between local and global axonal deformation, and their regulatory determinants [15–17]. For experimental models, macroscopic nerve fibers from animals (squid giant axons, guinea pig spinal cords and optic nerves, rat sciatic nerves, etc.) are widely adopted. Combined with uniaxial stretch loading and real-time electrophysiological recording, studies quantify correlations between injury severity and electrophysiological metrics such as compound action potential (CAP) amplitude, conduction velocity and latency, and further propose corresponding deformation injury thresholds [2,5,18–22]. Nevertheless, poor comparability persists between existing computational and experimental studies. First, they operate at disparate scales: high-fidelity axonal computational models focus on subcellular geometry and millisecond timescales, which cannot replicate macroscopic experimental boundary conditions or capture electrophysiological alterations minutes to days post-injury. Second, inconsistency exists in structural quantity: computational models typically only resolve a single node of Ranvier and adjacent segments, failing to reproduce collective physiological responses generated by abundant nerve fibers in experiments. Additionally, although empirical studies can phenomenologically quantify links between deformation parameters and functional electrophysiological deficits, neural functional injury precedes morphological damage. This indicates a lack of mechanistic interpretation bridging primary mechanical stimuli and neuronal electrophysiological outputs.

Neuronal electrophysiological function relies on ion channels. Early studies revealed that traumatic brain injury (TBI)-mimicking loading on neurons induces sustained axonal calcium influx that triggers secondary injury cascades, which can be blocked by the sodium channel blocker TTX, demonstrating the essential role of sodium channel leakage in this process [23–25]. Subsequent patch-clamp suction experiments on cells expressing Nav 1.4 and Nav 1.5 subtypes verified that membrane tension modulates sodium channel gating, yet the underlying mechanism remained unclear [26–28]. Wang et al. (2009) systematically conducted suction trauma and TBI-like stretch experiments on Nav 1.6-transfected cells [29], and discovered that membrane stretch shifts the steady-state activation and inactivation curves of sodium channels toward hyperpolarization, generating window currents near resting potential and elucidating the mechanism of sodium leakage. They defined this phenomenon as the “left-shift” injury mechanism of sodium channels, which lays a critical foundation for quantitatively characterizing neuronal electrophysiological injury using the Hodgkin-Huxley (HH) model.

Currently, there are two main pioneering explorations: first, the coupled left-shift damage model proposed by Boucher et al [30], which for the first time introduced Nav left-shift value and injury ratio as parameters into the calculation of Nav channel gating rate constants and Nav current, but the relationship between these two parameters and (axonal) deformation is not clarified in this model; second, the mechano-electrical coupling model of spinal cord injury by Jérusalem et al. [31], which pioneered integrating axonal deformation as injury into the reversal potentials and gating rates of sodium and potassium (Kv) channels, profoundly influencing subsequent peer studies on neuro-mechano-electrical injury modeling [32–34]. They referenced the experimental conditions reported by Shi and Whitebone [5], and compared the predicted CAP amplitudes from the model against experimental measurements. A notable limitation lies in the complete disappearance of CAP amplitudes under severe damage conditions, which deviates from experimental observations. This discrepancy potentially originates from the scale discrepancy: the model is constructed at the single‑axon scale, whereas the corresponding experiments adopt composite nerve bundles as test specimens. Severe damage to individual axons does not necessarily lead to total functional failure of the entire nerve bundle. Subsequently, Cinelli et al. developed a finite‑element nerve‑bundle model with four axons and innovatively adopted an electro‑thermal equivalent framework to realize the coupling of mechanical and electrophysiological injury [32]. Nevertheless, this model suffers from limited structural representativeness and neglects the intrinsic viscoelasticity of biological neurons in material properties, which fails to capture the rate‑dependent behavior of neural injury. García‑González et al. first proposed an energy‑based coupled damage model at the tissue scale [33]. Although this model yields reasonable damage predictions consistent with partial experimental data under quasi‑static and high‑strain‑rate conditions, its electrophysiological module built upon the FitzHugh‑Nagumo model cannot interpret the pathological mechanisms underlying ion‑channel injury. Moreover, its mechanical damage evolution is confined to the macroscopic level and incapable of characterizing microscale damage features. Given these drawbacks of existing models, improved modelling frameworks are urgently required. Such frameworks should enable reliable damage prediction as well as faithful reproduction of the relevant pathological mechanisms and micro‑damage evolution, so as to comprehensively interpret the electrophysiological functional injury induced by nerve bundle deformation.

Herein, building on the work of peers, we propose an improved statistical damage model that extends the mechano-electrical coupling theory describing neural deformation injury from a single axon to the quantitative level of nerve bundles. First, at the single‑axon level, we divide the Nav and Kv currents into damaged and undamaged phases according to the damage fraction. Coupled interactions between impaired Nav and Kv channels are postulated, and such interactions are represented by modifying the ionic reversal potentials and gating rate constants. Second, at the nerve bundle level, we assume that individual micro axons exhibit heterogeneous damage states, which follow a Weibull distribution; their collective effects can therefore be quantified via integral operations. Afterwards, classic experimental conditions were reconstructed to optimize model parameters and conduct error comparison. Finally, by examining the evolution of damage distribution curves under various loading conditions and at different time instants, we observe that the heterogeneity of micro-axon damage progressively intensifies as the overall macroscopic deformation‑induced damage level of the nerve bundle rises.

## 2. Results

As illustrated in Fig 1, the framework of the neural injury model proposed in this paper consists of four core components, namely the mechanical model, the fundamental electrophysiological model (Fig 1(a)), injury coupling at the single axon level (Fig 1(b)), and integral processing of injury distribution extended to the nerve bundle level (Fig 1(c)). Detailed derivations are presented in the Methods section. To quantify the severity of axonal injury, the microscale axonal strain *ε*_*ma*_ and the irreversible permanent damage strain *ε*_*D*_ need be calculated. In the research conducted by Jérusalem et al. (2014) [31], the injury magnitude of an individual axon can be derived from *ε*_*ma*_, and the degree of electrophysiological functional impairment can be evaluated via the response of compound action potentials (CAPs). Nevertheless, we consider that the damage magnitude of microscale axons within a nerve bundle, which is indicated by *ω*_*D*_, follows an inhomogeneous distribution, and necessitates characterization by a distribution function. The Weibull distribution is adopted herein, with its distribution mean *M* correlated to *ε*_*ma*_. Subsequent sections demonstrate the mechanical responses of axons under various loading conditions, the calibration outcomes of the parameter *M*, and the CAP predictions yielded by the model after all parameters are calibrated.

**Fig 1:**
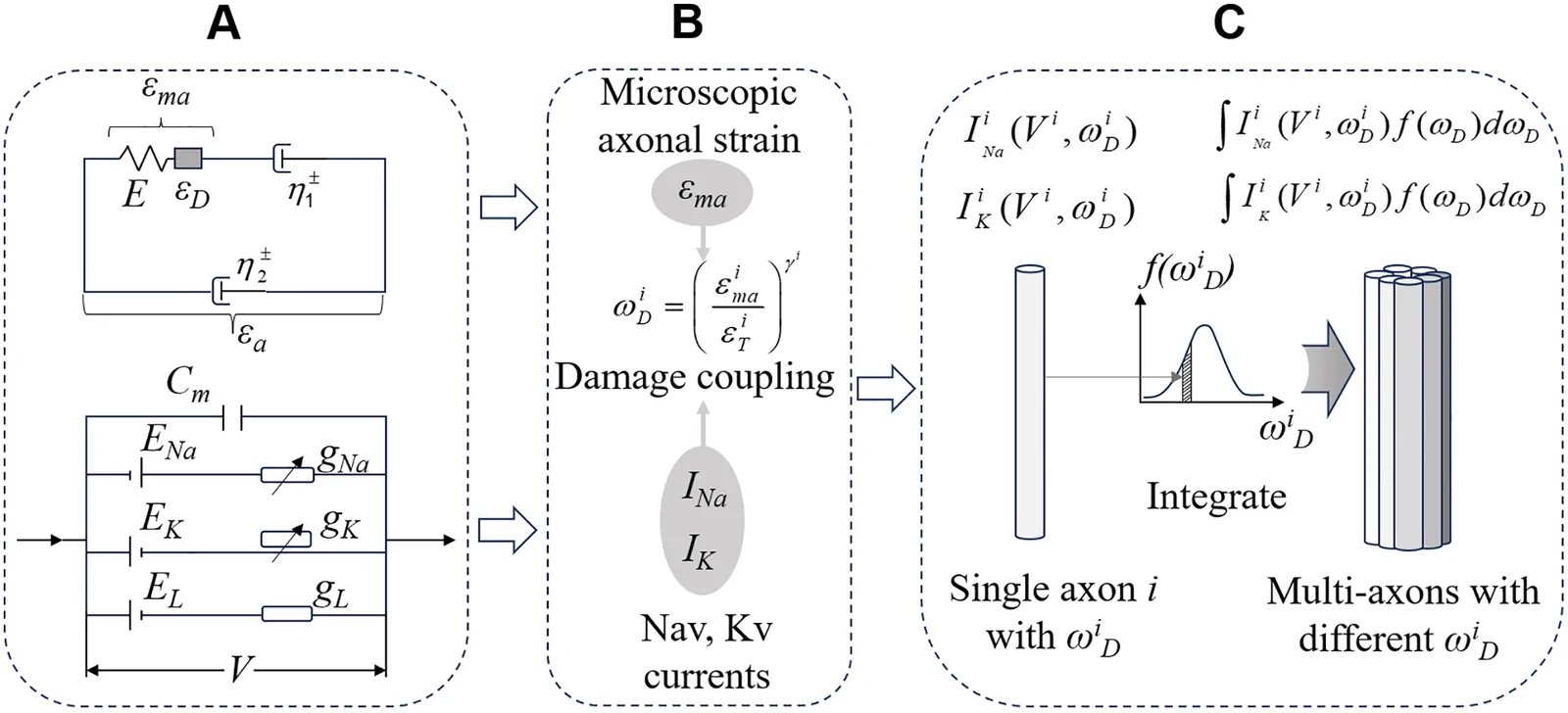
Overall framework of the damage model. **(a)** (Top) Mechanical constitutive model of the axon; (Bottom) Electrophysiological model of the axon. Among them, the macroscopic strain of the axon is characterized by the overall strain of the element *εₐ*, the microscopic strain by *ε_ma_*, and *ε*_*D*_ is the irreversible damage strain; the electrophysiological model adopts the Hodgkin-Huxley (HH) model; **(b)** The microscopic strain *ε_ma_* obtained from the mechanical model is incorporated as the damage parameter *ω*_*D*_ into the sodium (Nav) and potassium (Kv) currents of the HH model, realizing damage coupling at the single axon level; **(c)** Considering the microscopic heterogeneity within the nerve bundle, it is assumed that although the damage parameters *ω*_*D*_ of each microscopic axon are different, they all follow the Weibull distribution *f(ω_D_)*. Based on this, the Nav and Kv currents of all microscopic axons after damage can be integrated to obtain the Nav and Kv currents at the nerve bundle quantity level, thereby calculating the compound action potential (CAP).

### 2.1. The mechanical response of axons

The viscoelastic-plastic model proposed by Jérusalem et al. (2014) is adopted as the axonal mechanical model in this paper, with the classical injury experiments on spinal cord white matter strips reported by Shi and Whitebone (2006) referenced to define the loading conditions. Their work covers various loading conditions and comprehensively records the spinal electrophysiological responses at different time points after injury. In brief, the guinea pig spinal cord white matter strips were stretched to three different strain levels (“mild”, “moderate”, and “severe”, respectively) under two strain rates (“fast” and “slow”). Subsequently, the load was removed to allow free relaxation of the strips. The specific working condition parameters are listed in Table 1. The microscopic axonal strains *ε_ma_* under each working condition are shown in Fig 2(a), which is calculated via Equations (3)–(5). It can be seen that even when loaded to the same macroscopic strain level, due to the higher strain rate of the fast group, the corresponding microscopic axonal strains *ε_ma_* and the induced damage strains *ε*_*D*_ are generally higher than those of the slow group. As shown in Fig 2(b), the damage strains *ε*_*D*_ calculated by Equation (5) are ordered from smallest to largest as follows: slow-mild, slow-moderate, fast-mild, slow-severe, fast-moderate, and fast-severe. Among them, the microscopic axonal strains and damage strains of fast-mild and slow-severe are highly close, which also indicates that axonal damage is closely related to both the deformation amplitude and loading rate during loading.

**Table 1:** Working conditions for stretch in the work of Shi and Whitebone (2006).

Working conditions		Mild	Moderate	Severe
Strain amplitude (*ε*_*max*_)		0.25	0.5	1
Strain rate (*ε̇₀*)	fast	355 s^-1^	443 s^-1^	519 s^-1^
	slow	0.006 s^-1^	0.007 s^-1^	0.008 s^-1^

**Fig 2:**
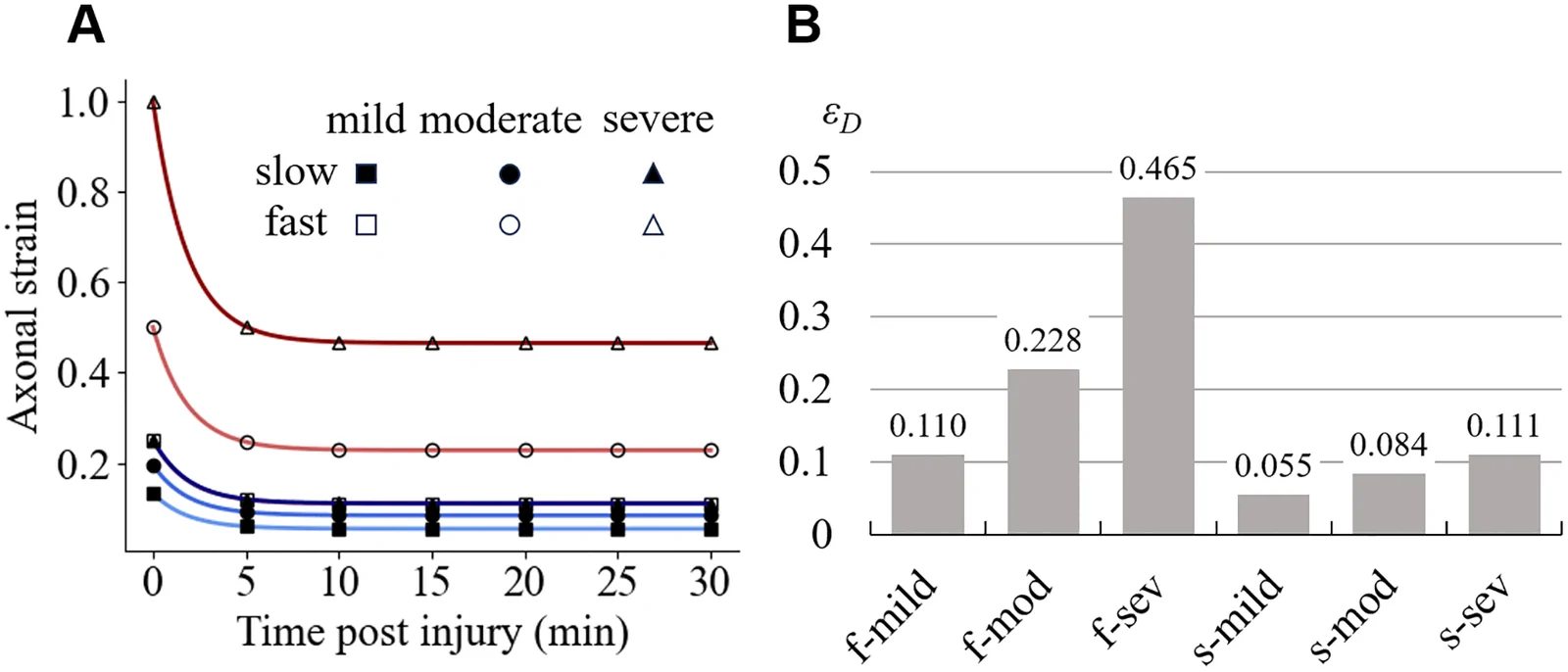
Calculation results of the axonal mechanical sub-model (a) Time-history curves of axonal microscopic strain *ε*_*ma*_ under each working condition: the red series with open symbols represent the fast group, and the blue series with solid symbols represent the slow group. The curves include time-recoverable viscoelastic strain and irrecoverable damage strain; **(b)** Irreversible damage strain *ε*_*D*_ generated in axons under each working condition.

### 2.2. The mean value of damage distribution

Following the calculation of the axonal strains, we further investigate the damage distribution of microscopic axons under various loading conditions. First, the remaining model parameters are determined in accordance with the procedures detailed in the Methods section. Subsequently, the distribution mean *M* corresponding to each loading condition is calibrated in two separate steps by minimizing the discrepancy between the predicted CAP responses of injured axons and experimental measurements. Fig 3(a) and 3(b) show the preliminarily calibrated *M* values corresponding to all working conditions listed in Table 1. The CAP recovery level calculated using these *M* values are presented in Fig 3(c), where the green curves represent the simulation results and the black curves represent the experimental results from the original work [5]. It can be seen that the two are highly consistent. Meanwhile, these fitted “optimal” *M* values exhibit a significant exponential decay trend over time, similar to the relaxation strain characteristics of axons. As the mean parameter of the injury distribution, *M* quantifies the overall extent of axonal damage and is hypothesized to hold a direct correlation with axonal deformation. Therefore, we fitted the *M* values based on Equations (3) and (18) to establish their correlation with axonal strain. The fitting results are shown by the dashed lines in Fig 3(a) and 3(b), and the fitting formula is as follows,

**Fig 3:**
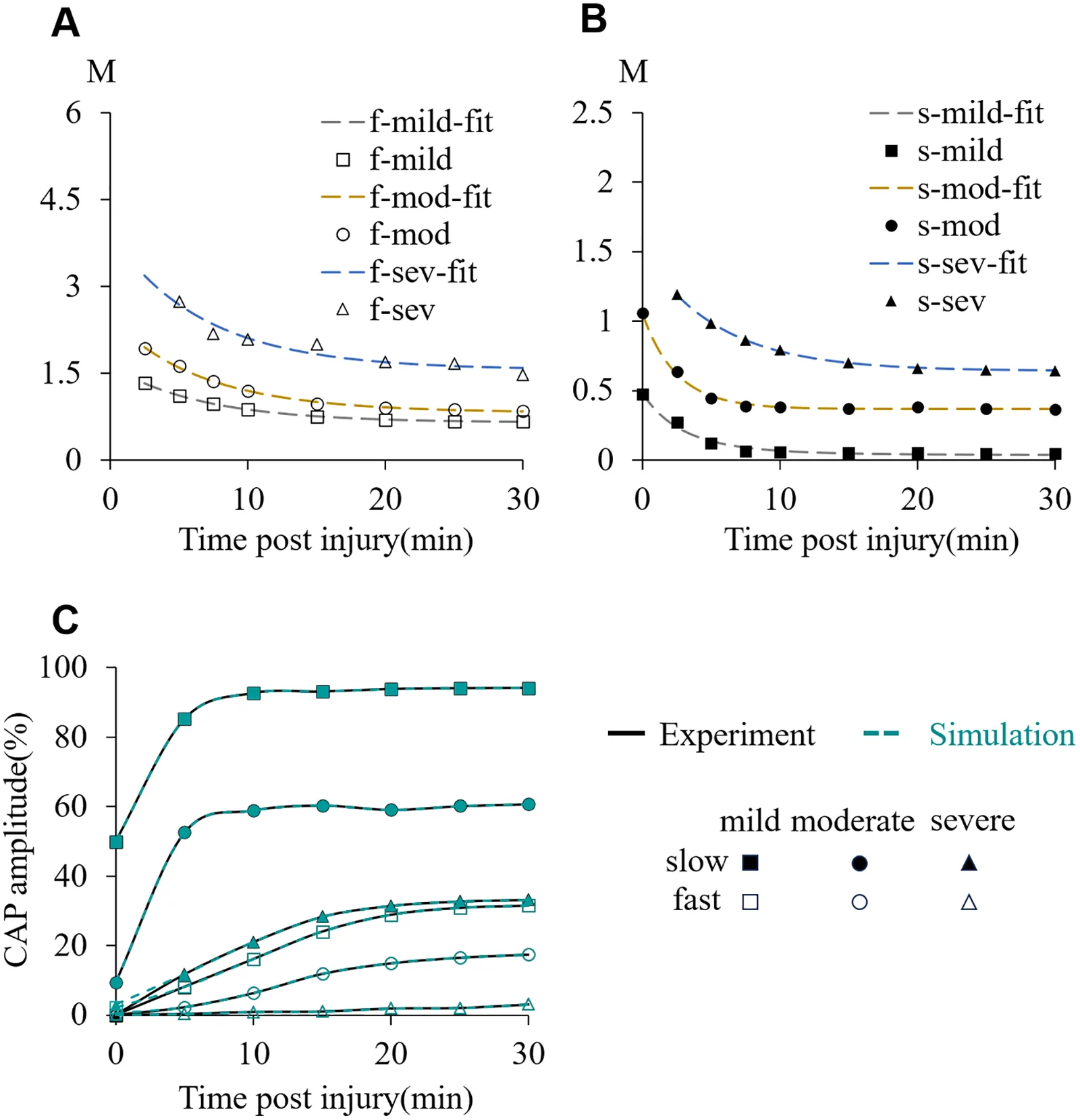
Calculation results of the preliminary calibrated “optimal” distribution mean *M* (a) Fast group (open symbols) and (b) slow group (solid symbols), showing that *M* exhibits a significant exponential decay trend over time; (c) Comparison between the CAP recovery levels calculated from these optimal *M* values (green) and the experiments by Shi and Whitebone (2006) (black), ensuring maximum consistency with the experimental results.


$$\begin{array}{c} M={\left(\frac{\varepsilon_{D}+(\varepsilon_{ma}^{*}-\varepsilon_{D})\text{exp}(-t/\tau )}{\varepsilon_{T}}\right)}^{\gamma } \end{array}$$ (1)

Where *τ* is the characteristic time for damage recovery, *ε*_*T*_ is the disability strain threshold, and *γ* is the sensitivity to deformation of the axon. Fitted parameters of Equation (1) are listed in Table 2.

**Table 2:** Fitted parameters for preliminarily calibrated *M* values in Equation (1).

Working conditions	Fast-mild	Fast-moderate	Fast-severe	Slow-mild	Slow-moderate	Slow-severe
*ε*_*T*_	0.162	0.264	0.324	0.173	0.188	0.170
*γ*	1.138	1.413	1.210	2.819	1.254	1.043
*τ* (min)	6.990	7.424	7.183	5.364	2.653	5.607

For the same type of nerve, its deformation tolerance threshold should be consistent, thus the threshold strain *ε*_*T*_ under each working condition should be a constant. As can be seen from Table 2, after excluding the maximum and minimum *ε*_*T*_ values, the remaining *ε*_*T*_ values under each working condition are relatively close. Based on this, we take the average value after excluding the extreme values as the fixed value of *ε*_*T*_ (approximately 0.1986), and substitute it back into Equation (1) to re-fit the *M* values for secondary calibration. The results are shown in Fig 4(a) and 4(b). The time constants *τ* and parameters *γ* of each working condition after secondary calibration are presented in Fig 4(c) and 4(d).

**Fig 4:**
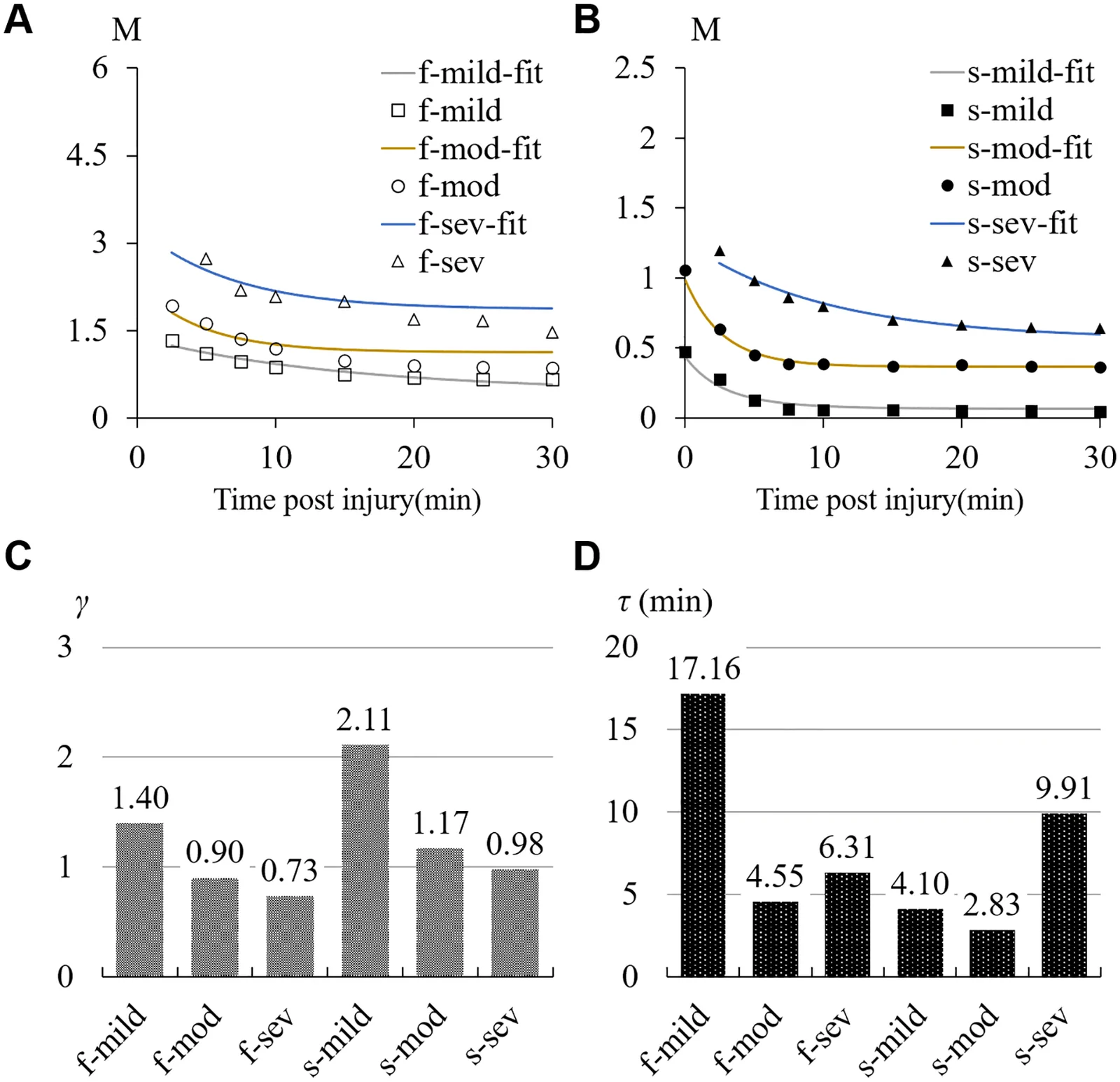
Secondary calibration results of distribution mean *M*, after determining the functional damage threshold *ε*_*T*_ = 0.1986 from the fitting parameters of the preliminary calibrated values and substituting it back into the fitting equation. **(a)**: Fast group and **(b)**: slow group; after refitting *M*, the distributions of (c) damage sensitivity *γ* and (d) characteristic recovery time τ under each working condition. Among them, the *γ* values of the fast group are significantly lower than those of the slow group, while the *τ* values are generally higher than those of the slow group.

### 2.3. CAP recovery level

The degree of CAP recovery calculated from the re-fitted *M* values after back-substitution is shown in Fig 5(a). It can be observed that although there is a deviation between the re-fitted results and the optimal case in Fig 3(c), they still maintain a good agreement with the experimental results. As the earliest proposed axon mechano-electrical coupling model, the original results of Jérusalem et al. [31] are also included in the comparison, as shown in Fig 5(b). The overall errors of the two models at different time points after injury and under various working conditions are presented in Fig 5(c). The error is defined as the root mean square error (RMSE), which is expressed in Equation (2),

**Fig 5:**
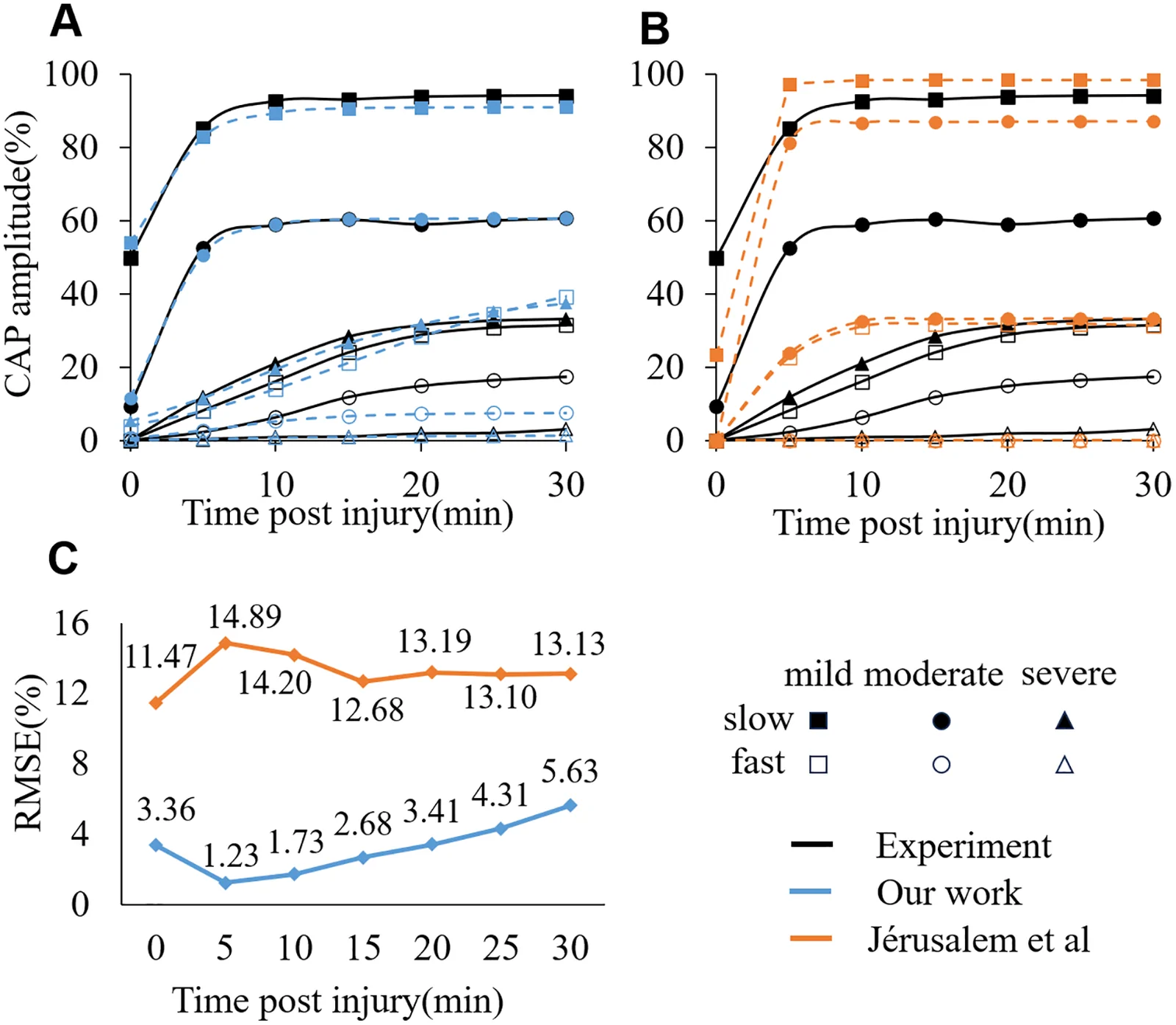
CAP recovery levels under each working condition and comparison with peer models (a) Comparison between CAP amplitudes calculated from the final fitted parameters after substituting back *M* values and experimental results; (b) Comparison between the original results of Jérusalem et al. (2014) and experimental results; (c) Comparison of overall errors between the present work, Jérusalem’s original study and experimental results at different post-injury times.


$$\begin{array}{c} RSME(\%)=\sqrt{\frac{\text{1}}{n}\sum_{i}^{n}({y^{sim}}_{i}-{y^{exp}}_{i}{)}^{\text{2}}} \end{array}$$ (2)

Here, *y* denotes the CAP recovery level obtained from simulations (sim) or experiments (exp), and the subscript *i* corresponds to different loading conditions at the same recovery time. The results indicate that although the error of our model increases at 20 minutes after injury, it still has a significantly better overall agreement effect.

As shown in Fig 6, in addition to the overall error, we also compared the error performance of our model and the one of Jérusalem et al. ‘s [31] under each specific working condition. Except for the fast-severe working condition, our model is closer to the experimental results for most of the time after injury. For the fast-severe working condition, since the axonal strain exceeds the functional damage threshold at all time points, the action potential amplitude calculated by the single-axon level Jérusalem et al.’s model is constantly 0 mV. In the experiment, the CAP amplitude measured for this working condition within 0-15 minutes after injury is also close to 0 mV, but a slight recovery occurs after 20 minutes. Therefore, the results of Jérusalem et al.’s model are more consistent with the experiment within the first 15 minutes, while the proposed model can more accurately characterize the recovery of CAP amplitude after 20 minutes.

**Fig 6:**
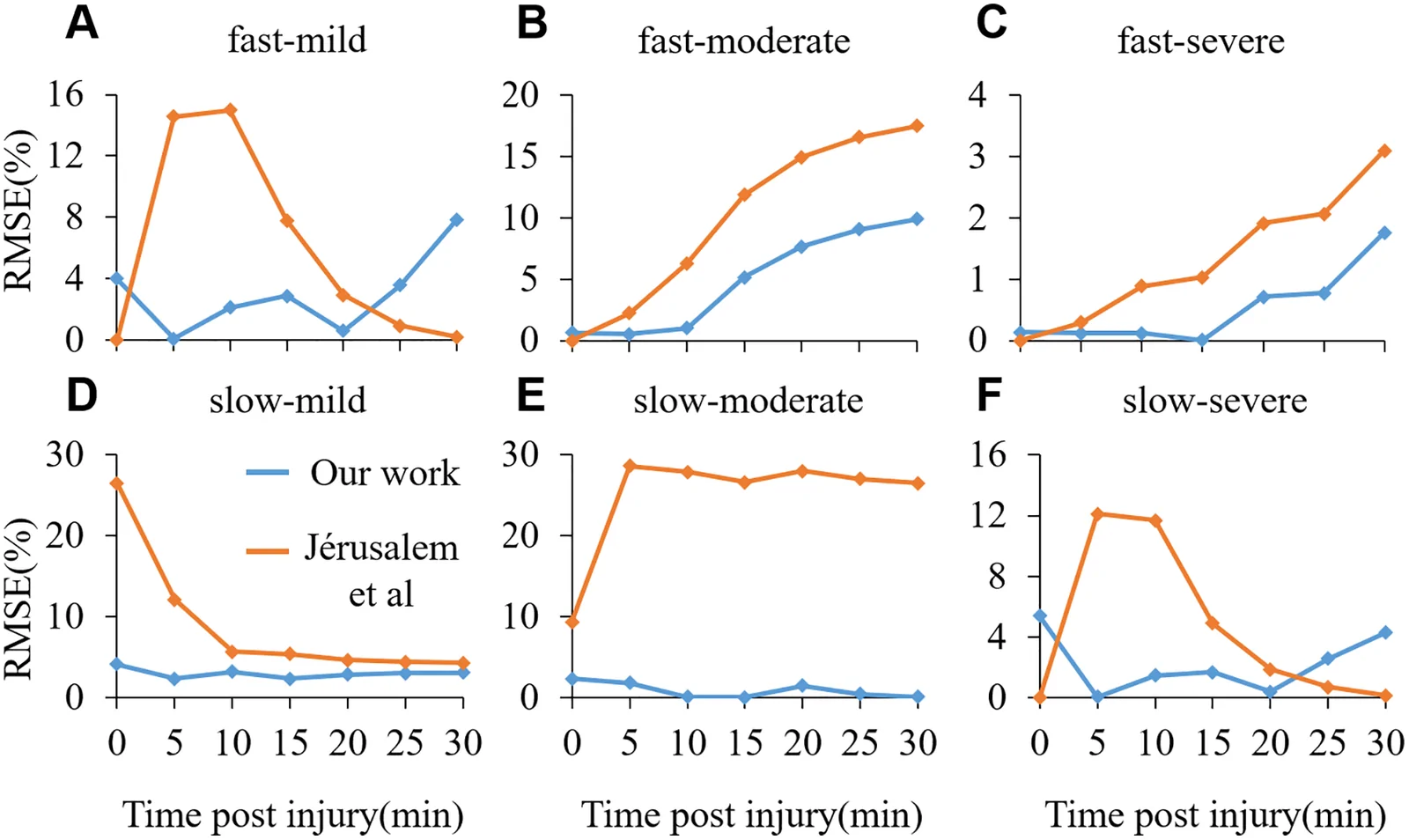
Error comparison between the present model, the original results of Jérusalem et al. (2014) and experimental results under different post-injury times for each working condition, where (a)–(c) correspond to the fast group and (d)–(f) to the slow group.

### 2.4. Damage distribution curves under individual working conditions

As shown in Fig 7, after determining all model parameters under each working condition, we can obtain the distribution curves of damage parameter *ω*_*D*_. The damage distribution curves of all working conditions exhibit similar variation trends with recovery time. Taking the fast-mild working condition as an example: at the instant after injury (0 minute), the distribution curve is located in the region with a higher degree of damage and has a wider distribution range, which is consistent with the characteristic of generally larger axonal strains in the early relaxation stage; as time progresses to 30 minutes, the distribution curve shifts to the region with a lower degree of damage and becomes more concentrated, corresponding to the state where axonal strains gradually approach the irreversible damage strain in the late relaxation stage.

**Fig 7:**
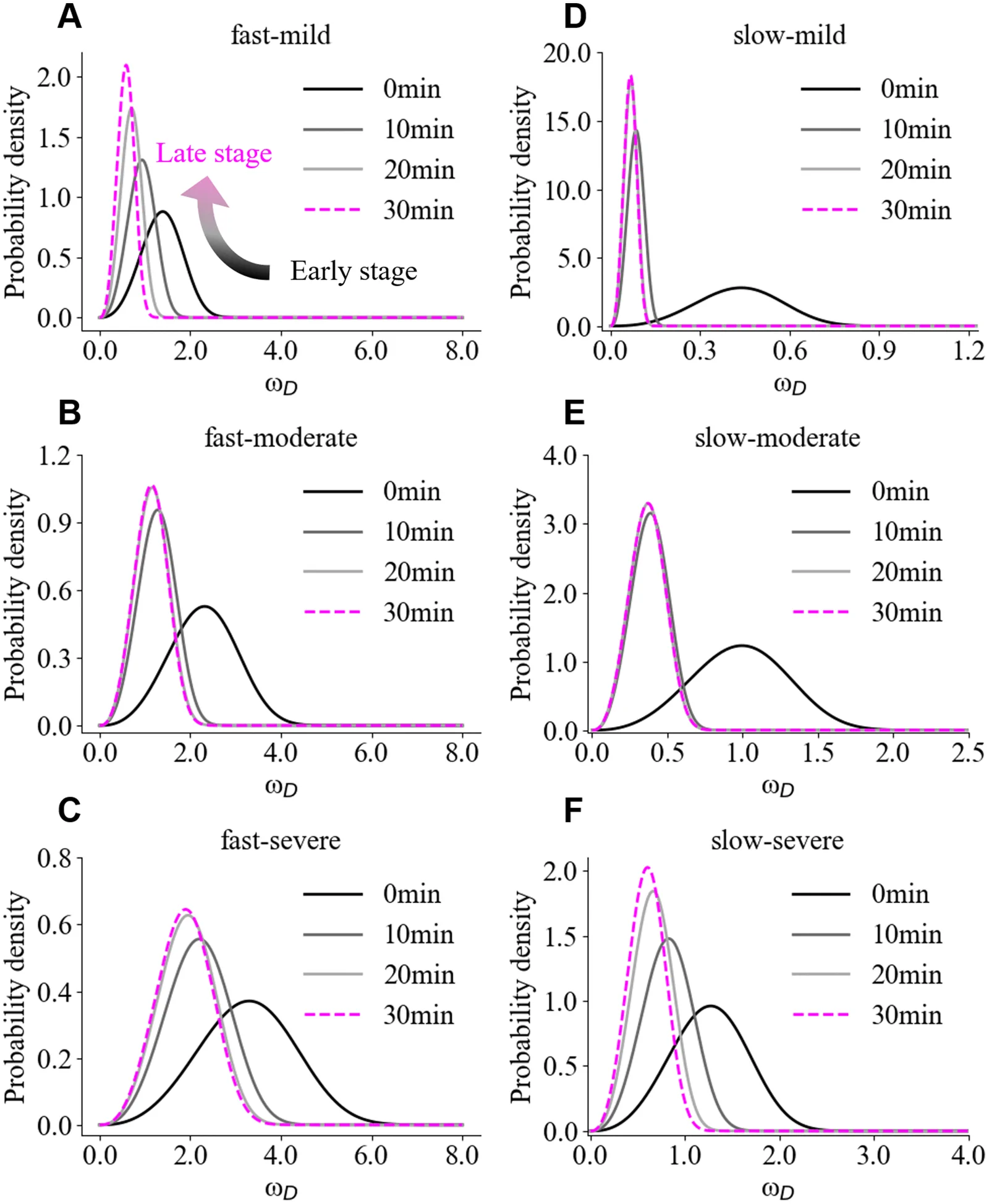
Temporal changes of damage distribution curves under different working conditions. **(a)–(c)** correspond to the fast group, and **(d)–(f)** to the slow group. For readability, 4 time points are selected from all 7 post-injury time points, and the distribution curves at 30 min for each working condition are highlighted in magenta. It can be seen that in the early post-injury stage, the distribution curves are relatively scattered with a large mean; as time progresses, the mean decreases and the distribution curves gradually become concentrated.

As shown in Fig 8, under different recovery times, the damage distribution curves corresponding to working conditions of different damage levels also exhibit similar variation trends. For different working conditions, with the increase of the damage strain *ε*_*D*_, the positions of the corresponding distribution curves gradually shift toward the direction of increasing *ω*_*D*_, and the distribution range gradually widens.

**Fig 8:**
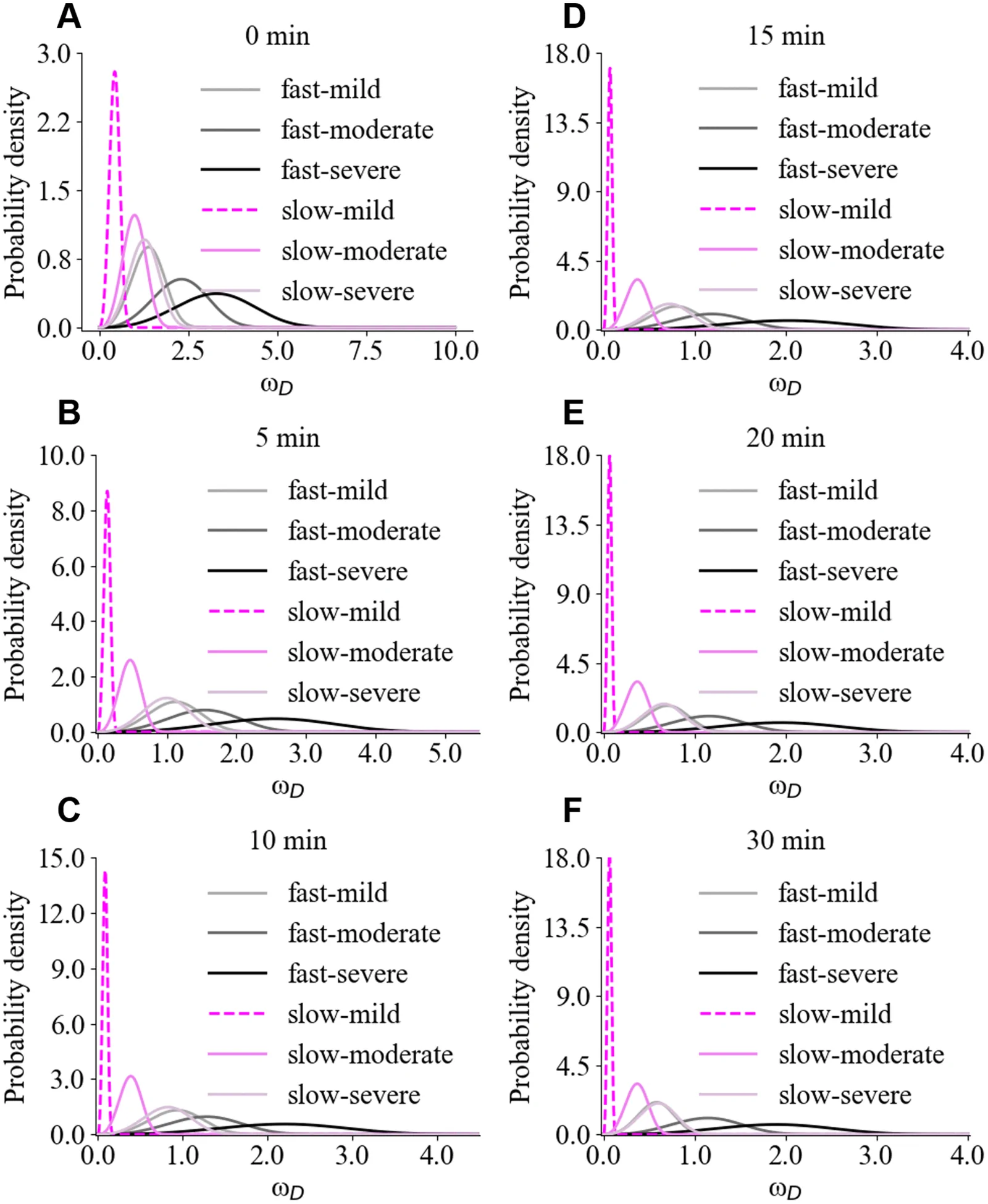
Changes of damage distribution curves with working conditions under different recovery times. **(a)–(c)** correspond to 0–10 min post-injury, and **(d)–(f)** to 15–30 min post-injury. For readability, the slow-mild group with the mildest damage is highlighted in magenta. It can be observed that at different times, the distribution curves of the slow group (with milder damage) are generally more concentrated than those of the fast group.

## 3. Discussion

This paper proposes a statistical damage model suitable for describing the deformation damage of nerve bundles. Its core lies in continuously treating a large number of microscopic axons in the nerve bundle and characterizing the damage at the nerve bundle quantity level through the integration of the damage probability distribution. The Weibull distribution is selected as the probability distribution form due to its high flexibility, which can be adjusted through parameters to be applied to failure rate analysis in different scenarios [35–38]. The three parameters associated with the Weibull distribution are the shape parameter *k*, scale parameter *λ*, and distribution mean *M*. Among them, *k* and *λ* are correlated with *M* through Equation (25), and *M* is significantly related to axonal strain. Therefore, there is only one independent parameter to be determined for the probability distribution under all working conditions. Since the scale parameter *λ* determines the range and position of the distribution, and its physical meaning is similar to that of the distribution mean *M*, to avoid redundant definition of physical concepts, we make *M* to be the finally determined distribution parameter after a priori determination of *k* = 3.5. The premise for choosing this *k* value is the assumption that the micro-axon injury amount caused by deformation is concentrated around the distribution mean. Although the fitting results under the current parameter combination can well agree with the experimental results of peers, finding substantial experimental evidence in the future remains an important research direction.

One of the core aspects of the modeling in this paper is the left-shift injury mechanism of sodium channels. The fitted value of the injury translation coefficient *C* for gating rates in Equation (22) is 0.85. Substituting the reference values of reversal potentials for sodium and potassium channels from Table 3, the maximum left-shift value of Nav is calculated as 30.45 mV, which is close to or covers the limit values reported in previous studies on suction experiments of various sodium channel subtypes. In the work of [29], the maximum left-shift value of Nav 1.6 was reported to be approximately 35 mV, where the suction negative pressure applied to the cells reached -45 mmHg—near the limit of cell membrane rupture. This value was also adopted as the upper limit of left shift in the modeling work of Boucher et al. [30]. Beyder et al. [39] reported a maximum left-shift value of 26 mV in suction experiments on HEK cells transfected with Nav 1.5. Extrapolating from their conclusions, a left-shift value of 30 mV corresponds to a suction negative pressure of approximately -43 mmHg, which exceeds the stability limit of the patch structure (about -30 mmHg). Two earlier studies reported maximum left-shift values of 4 mV (Nav 1.5) and 20 mV (Nav 1.4), respectively [27,28]. Collectively, the maximum left-shift value (30.45 mV) reported in this paper sufficiently reflects extreme injury conditions while avoiding exceeding the experimentally supportable range, thus possessing clear biophysical rationality.

**Table 3:** Electrophysiological parameters [55] and simulation settings.

Parameter	Value
Leak reversal potential (*E*_*L*_)	-54.4 mV
Kv reversal potential (*E*_*K0*_)	-77.5 mV
Nav reversal potential (*E*_*Na0*_)	49.5 mV
Leak conductance (*g*_*L*_)	0.3 mS/ cm^2^
Maximal Kv conductance (${\overset{―}{g}}_{K}$)	36 mS/ cm^2^
Maximal Nav conductance (${\overset{―}{g}}_{Na}$)	120 mS/ cm^2^
Membrane capacity density (*C*_*m*_)	1 uF/ cm^2^
External current (*I*_*ext*_)	50 uA/ cm^2^
Time step	0.005 ms
Simulation duration	120 ms
Integral step for *ω*_*D*_	0.001

A notable phenomenon observed during the simulation in this paper is the spontaneous spiking behavior of damaged neurons. As shown in Fig 9, taking the fast-mild-30min working condition as an example, when the translation coefficient *C* of the sodium and potassium rate constant is 0.85 (determined by fitting), there is basically no spontaneous spiking phenomenon. However, when the value of *C* is smaller, ectopic excitatory oscillations of the membrane potential still occur even without external current. This ectopic spontaneous spiking phenomenon is consistent with the observations reported in previous experimental and simulation studies [40,41], namely that the excitability level of the cortical network is abnormally elevated following traumatic brain injury, which may be associated with the ionic mechanisms underlying epileptic seizures [42]. The ectopic firing caused by the left-shift injury of sodium channels and the underlying dynamic bifurcation mechanism have been fully discussed in the studies by Boucher and his colleagues [30,43,44]. Recently, the role of potassium channels in ectopically excited neural activities has also been gradually revealed. In some studies using ligation or transection as mechanical injury methods, phenomena such as increased proportion of spontaneous neuronal discharge, decreased discharge threshold, and enhanced subthreshold oscillation behavior have been observed [45–48]. Moreover, the increased pain sensitivity accompanied by these abnormally excited neural activities is also associated with the downregulation of the expression of certain potassium channel subtypes [49,50]. In the future clinical treatment of epileptic or neuropathic pain, specific interventions targeting ion channels may become a potential therapeutic strategy.

**Fig 9:**
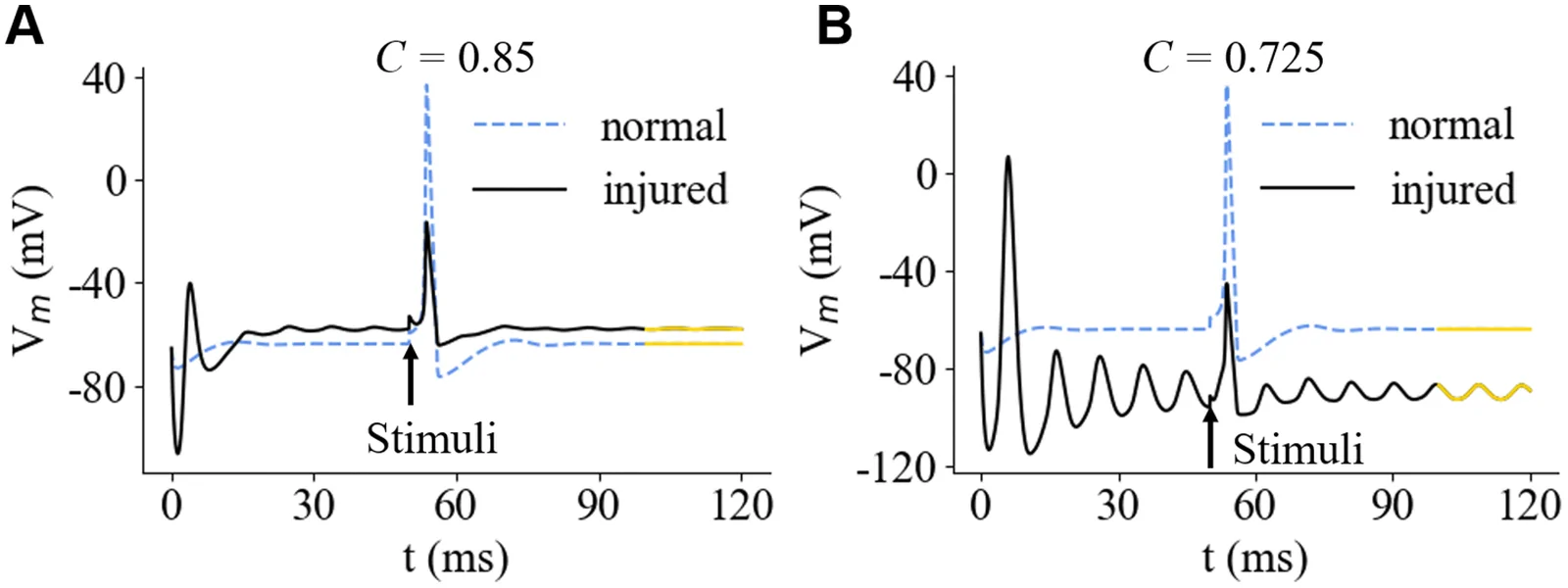
Schematic diagram of spontaneous membrane potential oscillation under the fast-mild-30min working condition as an example, with different translation coefficients *C* of sodium and potassium channels. **(a)** When *C* is 0.85 (determined by fitting), there is basically no spontaneous spiking phenomenon, and CAP is only generated after applying a pulse stimulus (marked by the black arrow); **(b)** When *C* takes a smaller value, self-discharge is more obvious, indicating that this phenomenon is related to both sodium and potassium channels. Since the resting potential adopts the average value of the membrane potential in the 20 ms before the end of the simulation (as shown by the yellow line), excessively significant spontaneous spiking will lead to a large error in CAP calculation.

Combining the variation trends of the distribution curves shown in Figs 7 and 8, it can be concluded that the greater the deformation damage of the macroscopic nerve bundle, the higher the overall damage level of the internal microscopic axons and the more significant the damage inhomogeneity; conversely, the smaller the macroscopic damage, the lower the overall axonal damage and the more uniform the damage. This damage inhomogeneity may stem from two factors: first, the multi-level organizational structure of the nerve bundle (e.g., the perineurium and epineurium with tensile resistance and buffering effects, and the nerve fascicles that branch, reorganize, and have uneven orientations along the length direction). The heterogeneity of these connective tissues allows microscopic axons to retain partial functions under supraphysiological threshold loads [51]; second, the loading condition of the reference experiment—Shi and Whitebone [5] adopted a three-point bending-like loading method (the spinal cord strip was fixed at both ends and the middle section was suspended, with loading achieved by stretching the center of the suspended section), resulting in the actual deformation of axons being related to their positions. This conclusion has been confirmed by their earlier experimental studies: under tensile conditions, the number of axons labeled with horseradish peroxidase (HRP) on the outer side of the spinal cord strip was significantly more than that on the inner side, indicating more severe damage to the outer axons; under compressive injury conditions, there were also significant differences in the distribution positions of labeled axons in the same cross-section, both proving the inhomogeneity of microscopic damage inside the nerve bundle [10].

By comparing the calculated damage strains *ε*_*D*_ under each working condition in Fig 2(b) with the fitted axonal deformation sensitivity *γ* in Fig 4(c), an obvious negative correlation is observed between the two. As shown in Fig 10, further fitting results indicate a significant negative exponential correlation between them. Working conditions with smaller *ε*_*D*_ are usually caused by low strain rate loading, and from the results of the two strain rates in Fig 4(c), it can be seen that axons exhibit higher sensitivity to deformation under low strain rate loads. This sensitivity is not necessarily related to mechanical strength but may be associated with the physiological functions of axons. Pfister et al. [52] conducted extreme growth studies on axons of rat dorsal root neurons and found that at a daily stretching rate of 1–8 mm, the growing axons not only maintained the integrity of microtubules and neurofilaments but also had an average cross-sectional area increased by approximately 35% compared with unstretched axons. Sousa et al. [53] demonstrated that a daily stretching rate of 1 mm could induce the translocation of Yes-associated protein (YAP) to the nucleus in embryonic dorsal root ganglion (DRG) neurons and promote the MARCKS-dependent cell membrane integration process. The above research results indicate that physiological processes such as axonal growth and remodeling exhibit highly adaptive and plastic responses to low-speed mechanical stretching.

**Fig 10:**
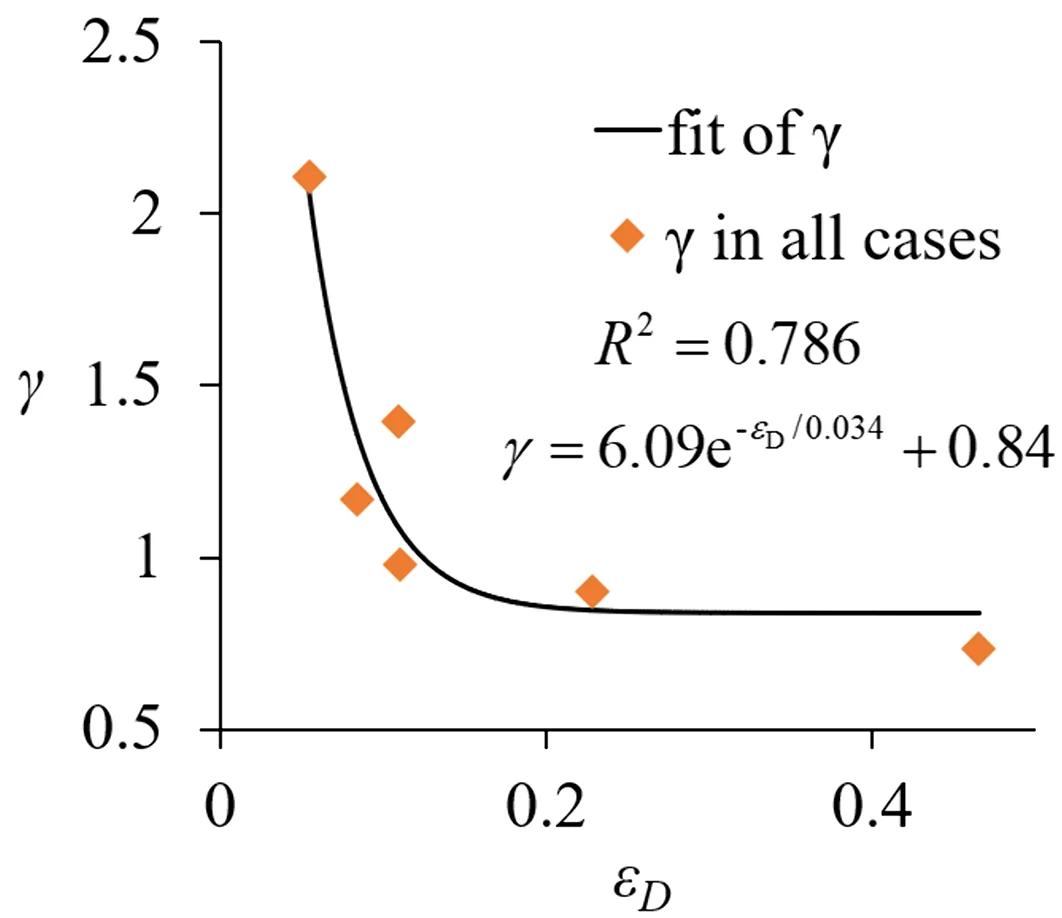
Variation of axonal deformation sensitivity *γ* and damage strain *ε*_*D*_ under different working conditions, showing a negative exponential correlation.

One major limitation of this study is that model calibration and validation rely entirely on the experimental CAP dataset reported by Shi and Whitebone (2006). Although this dataset records axonal injury evolution at multiple time points and provides a solid basis for damage‑kinetics analysis, its strain‑rate loading range is relatively narrow (355‑519 s^−1^ for the fast group and 0.006‑0.008 s^−1^ for the slow group). Consequently, the distribution of damage strain *ε*_*D*_ remains concentrated across different loading conditions, as illustrated in the histogram of Fig 2(b). This may degrade the identifiability and fitting robustness of rate‑sensitive parameters strongly coupled with *ε*_*D*_, namely *τ*, *ε*_*T*_ and *γ* in Equation (1). Accordingly, the extrapolation capability of the current model toward extreme strain‑rate conditions (e.g., > 1000 s^−1^) requires further validation. To address these limitations, future work will be carried out in two respects. First, axonal stretch‑injury experiments covering a broader strain‑rate spectrum (from quasi‑static 1 s^−1^ up to high strain rates >1000 s^−1^) will be performed to enrich the rate‑dependent damage database. Second, cross‑validation against independent electrophysiological measurements will be pursued, such as the conduction velocity and latency of action potential. These datasets can offer independent constraints beyond CAP amplitudes, enabling more rigorous assessment of the predictive performance of this coupling model and further extending its applicability across diverse injury scenarios

Another unresolved issue is that the role of the sodium-potassium pump is not considered to simplify the model. In the study by Boucher et al. [30], the sodium-potassium pump can perform compensatory regulation on ion leakage caused by sodium channel damage and regulate neuronal excitability by altering the concentration gradients of sodium and potassium ions. Future research will incorporate this mechanism to further analyze the disruption of ion homeostasis balance and adenosine triphosphate (ATP) depletion at the nerve bundle level induced by damage. Furthermore, it is observed that the characteristic recovery time of axons exhibits a non-monotonic relationship with the damage strain under each working condition. At the same macroscopic strain level, the groups with higher strain rates show longer recovery times. This may be partly attributed to the insufficient accuracy of the aforementioned fitted parameter. On the other hand, for viscoelastic materials, different initial strains during the relaxation stage lead to differences in the measured characteristic times. However, as analyzed earlier, axons have different physiological sensitivities to different strain rates, and this difference may also contribute to the variation in characteristic recovery times. The underlying mechanism requires further clarification in future research.

## 4. Methods

As shown in Fig 1, the axonal damage model framework proposed in this paper mainly consists of four parts: a mechanical model, a basic electrophysiological model (Fig 1(a)), injury coupling at the single axon level (Fig 1(b)), and the integral processing of injury distribution extended to the nerve bundle level (Fig 1(c)). The referenced working conditions and calculation methods are also provided below.

### 4.1. Mechanical sub-model

Recent studies have shown that most living cells exhibit irreversible plastic deformation after the removal of mechanical loads, which originates from bond ruptures within the cytoskeleton [54]. Therefore, in the absence of more experimental evidence, the axon mechanical model adopted in this paper still follows the viscoelastic-plastic model from the work of [31]. Specifically, as shown in Fig 1(a), the mechanical behavior of the Ranvier’s node is modeled as a parallel component of a Maxwell element and a damper, which is used to characterize the microscopic axial stiffness of the axon, the viscosity of tissue active molecule reorganization, and the viscous molecular cross-linking with the surrounding tissue structure. The macroscopic axon strain is given by *εₐ*, and the microscopic Ranvier node strain is given by *ε*_*ma*_. Since most experiments perform electrophysiological measurements only after axonal stretch injury [5,10,22], we mainly focus on the relaxation behavior of axons. When the tensile strength exceeds the damage stress threshold, the microscopic relaxation strain of the axon during unloading is given by,


$$\begin{array}{c} \varepsilon_{ma}=(\varepsilon_{ma}^{*}-\varepsilon_{D})e^{-t/\tau^{-}}+\varepsilon_{D} \end{array}$$ (3)

Where ε^*^_ma_ is the microscopic axonal strain at the start of unloading, and *ε*_*D*_ is the plastic damage strain during unloading, which is specifically given by the following equations:


$$\begin{array}{c} \varepsilon_{ma}^{*}=[\sum (\text{1}+\alpha )-\tau^{*}{\dot{\varepsilon }}_{\text{0}}]e^{-(\frac{\varepsilon_{\text{max}}}{{\dot{\varepsilon }}_{\text{0}}}-t^{D})/\tau^{*}}+(\tau^{*}{\dot{\varepsilon }}_{\text{0}}-\alpha \sum ) \end{array}$$ (4)

$$\begin{array}{c} \varepsilon_{D}=(\tau^{+}\alpha {\dot{\varepsilon }}_{\text{0}}-\alpha \sum )(\text{1}-e^{-(\frac{\varepsilon_{\text{max}}}{{\dot{\varepsilon }}_{\text{0}}}-t^{D})/\tau^{*}}) \end{array}$$ (5)

Here, *ε̇₀* represents the strain rate during loading, *ε*_*max*_ represents the maximum strain level reached during loading, and *t*^*D*^ represents the moment when the damage strain occurs, which is given by,


$$\begin{array}{c} t^{D}=-\tau^{+}ln(\text{1}-\frac{\sum }{\tau^{+}{\dot{\varepsilon }}_{\text{0}}}) \end{array}$$ (6)

The detailed values of mechanical sub-model parameters are shown in Table 4.

**Table 4:** Parameters of mechanical sub-model from [31].

Parameter	Value
*α*	0.9
*Σ*	0.018
*τ* ^*+*^	18.08s
*τ* ^*-*^	111.5s
*τ* ^*^	34.352s

### 4.2. Electrophysiological basic model

Previous studies have demonstrated that nodes of Ranvier constitute high-risk sites for axonal injury following DAI [7,23], whose scale is generally much smaller than that of the myelin segments in the internodal regions. Therefore, we do not consider the distal conduction of axons, but focus mainly on the membrane potential dynamics at the nodes, which is described by the Hodgkin-Huxley (HH) model [55], as schematically shown in Fig 1(b). The governing equation for the transmembrane potential *V* is given by the following equation,


$$\begin{array}{c} \frac{dV}{dt}=\frac{\text{1}}{C_{m}}[I_{ext}-g_{L}(V-E_{L})-{\overset{―}{g}}_{Na}m^{\text{3}}h(V-E_{Na})-{\overset{―}{g}}_{K}n^{\text{4}}(V-E_{K})] \end{array}$$ (7)

Where *C*_*m*_ is the membrane capacitance density, *I*_*ext*_ is the applied current, *g*_*L*_ is the leakage channel conductance, ${\overset{―}{g}}_{Na}$ and ${\overset{―}{g}}_{K}$ are the maximum conductance of the Nav channel and Kv channel respectively, *E*_*Na*_ and *E*_*K*_ are the corresponding reversal potentials, *m* and *h* are the activation and inactivation variables of the Nav channel, and *n* is the activation variable of the Kv channel. Their evolution rule is as follows,


$$\begin{array}{c} \frac{dx}{dt}=\alpha_{x}(\text{1}-x)-\beta_{x}x,\begin{array}{cc} & x\to m,h,n \end{array} \end{array}$$ (8)

And the evolution rate constants *α*_*x*_ and *β*_*x*_ of these gate variables are respectively given by [55],


$$\begin{array}{c} \alpha_{m}=\text{0}.\text{1}\frac{\left(V+\text{40}\right)}{\text{1}-\text{exp}(-\frac{V+\text{40}}{\text{10}})} \end{array}$$ (9)

$$\begin{array}{c} \beta_{m}=\text{4}\text{exp}(-\frac{V+\text{6}\text{5}}{\text{1}\text{8}}) \end{array}$$ (10)

$$\begin{array}{c} \alpha_{h}=\text{0}.\text{07exp}(-\frac{V+\text{65}}{\text{20}}) \end{array}$$ (11)

$$\begin{array}{c} \beta_{h}=\frac{\text{1}}{\text{1}+\text{exp}(-\frac{V+\text{3}\text{5}}{\text{1}\text{0}})} \end{array}$$ (12)

$$\begin{array}{c} \alpha_{n}=\text{0}.\text{01}\frac{\left(V+\text{55}\right)}{\text{1}-\text{exp}(-\frac{V+\text{55}}{\text{10}})} \end{array}$$ (13)

$$\begin{array}{c} \beta_{n}=\text{0}.\text{1}\text{2}\text{5}\text{exp}(-\frac{V+\text{5}\text{5}}{\text{8}\text{0}}) \end{array}$$ (14)

Among them, the units of membrane potential in Equations (9)–(14) are all mV, and the time units are all ms. The referred value of each parameter in the electrophysiological model is shown in Table 3.

### 4.3. Coupling method at the single axon level by Jérusalem et al

Previous studies have shown that in addition to Nav channels, deformation can also cause leakage damage to 4-aminopyridine (4-AP)-sensitive Kv channels [56,57]. Considering the leakage damage of ion channels, their ability to maintain the ion concentration difference across the cell membrane will weaken as the damage degree increases, and the corresponding reversal potential will gradually approach 0 mV. Therefore, in the work of Jérusalem et al. (2014) [31], both the Nav and Kv reversal potentials (*E*_*Na*_ and *E*_*K*_) are expressed in a form that decreases gradually with the increase of membrane strain (*ε*_*m*_). Here, membrane strain is defined as the reduction in axon radius, which is derived from the constant volume condition,


$$\begin{array}{c} \text{1}+\varepsilon_{m}=\sqrt{\text{1}+\varepsilon_{ma}} \end{array}$$ (15)

$$\begin{array}{c} \begin{array}{c} E_{Na}=\left\{ \begin{array}{c} E_{Na\text{0}}\left[\text{1}-{\left(\frac{\varepsilon_{m}}{\varepsilon_{T}}\right)}^{\gamma }\right],\varepsilon_{m}<\varepsilon_{T}\;\\ \text{0}\;,\varepsilon_{m}\geq \varepsilon_{T} \end{array}\right.\;\\ E_{K}=\left\{ \begin{array}{c} E_{K\text{0}}\left[\text{1}-{\left(\frac{\varepsilon_{m}}{\varepsilon_{T}}\right)}^{\gamma }\right],\varepsilon_{m}<\varepsilon_{T}\\ \text{0}\;,\varepsilon_{m}\geq \varepsilon_{T} \end{array}\right.\; \end{array} \end{array}$$ (16)

Where *E*_*Na0*_ and *E*_*K0*_ are the reference values of the reversal potentials for Nav and Kv, *ε*_*T*_ is the axonal strain damage threshold, and *γ* is the sensitivity of axons to deformation. For the gating rate constants (*α*_*m*_, *α*_*h*_, *α*_*n*_, *β*_*m*_, *β*_*h*_, and *β*_*n*_), in the work of Jérusalem et al. (2014), they proposed that these rate constants implicitly incorporate the contributions of *E*_*Na0*_ and *E*_*K0*_. Therefore, Equations (9)–(14) are also expressed in a form related to *ε*_*m*_, as shown by Equation (17),


$$\begin{array}{c} \begin{array}{c} \alpha_{m}(V,\varepsilon_{m})=\left\{ \begin{array}{c} \text{0}.\text{1}\frac{\left(V+(\frac{\varepsilon_{m}}{\varepsilon_{T}}{)}^{\gamma }E_{Na\text{0}}+\text{4}\text{0}\right)}{\text{1}-\text{exp}(-\frac{V+(\frac{\varepsilon_{m}}{\varepsilon_{T}}{)}^{\gamma }E_{Na\text{0}}+\text{4}\text{0}}{\text{1}\text{0}})},\\ \text{0}.\text{1}\frac{\left(V+E_{Na\text{0}}+\text{4}\text{0}\right)}{\text{1}-\text{exp}(-\frac{V+E_{Na\text{0}}+\text{4}\text{0}}{\text{1}\text{0}})},\varepsilon_{m}\geq \varepsilon_{T} \end{array}\right.\;\\ \beta_{m}(V,\varepsilon_{m})=\left\{ \begin{array}{c} \text{4}\text{exp}(-\frac{V+(\frac{\varepsilon_{m}}{\varepsilon_{T}}{)}^{\gamma }E_{Na\text{0}}+\text{6}\text{5}}{\text{1}\text{8}}),\\ \text{4}\text{exp}(-\frac{V+E_{Na\text{0}}+\text{6}\text{5}}{\text{1}\text{8}}),\varepsilon_{m}\geq \varepsilon_{T} \end{array}\right.\;\\ \alpha_{h}(V,\varepsilon_{m})=\left\{ \begin{array}{c} \text{0}.\text{0}\text{7}\text{exp}(-\frac{V+(\frac{\varepsilon_{m}}{\varepsilon_{T}}{)}^{\gamma }E_{Na\text{0}}+\text{6}\text{5}}{\text{2}\text{0}}),\\ \text{0}.\text{0}\text{7}\text{exp}(-\frac{V+E_{Na\text{0}}+\text{6}\text{5}}{\text{2}\text{0}}),\varepsilon_{m}\geq \varepsilon_{T} \end{array}\right.\;\\ \beta_{h}(V,\varepsilon_{m})=\left\{ \begin{array}{c} \frac{\text{1}}{\text{1}+\text{exp}(-\frac{V+(\frac{\varepsilon_{m}}{\varepsilon_{T}}{)}^{\gamma }E_{Na\text{0}}+\text{3}\text{5}}{\text{1}\text{0}})},\\ \frac{\text{1}}{\text{1}+\text{exp}(-\frac{V+E_{Na\text{0}}+\text{3}\text{5}}{\text{1}\text{0}})},\varepsilon_{m}\geq \varepsilon_{T} \end{array}\right.\;\\ \alpha_{n}(V,\varepsilon_{m})=\left\{ \begin{array}{c} \text{0}.\text{0}\text{1}\frac{\left(V+(\frac{\varepsilon_{m}}{\varepsilon_{T}}{)}^{\gamma }E_{K\text{0}}+\text{5}\text{5}\right)}{\text{1}-\text{exp}(-\frac{V+(\frac{\varepsilon_{m}}{\varepsilon_{T}}{)}^{\gamma }E_{K\text{0}}+\text{5}\text{5}}{\text{1}\text{0}})},\\ \text{0}.\text{0}\text{1}\frac{\left(V+E_{K\text{0}}+\text{5}\text{5}\right)}{\text{1}-\text{exp}(-\frac{V+E_{K\text{0}}+\text{5}\text{5}}{\text{1}\text{0}})},\varepsilon_{m}\geq \varepsilon_{T} \end{array}\right.\;\\ \beta_{n}(V,\varepsilon_{m})=\left\{ \begin{array}{c} \text{0}.\text{1}\text{2}\text{5}\text{exp}(-\frac{V+(\frac{\varepsilon_{m}}{\varepsilon_{T}}{)}^{\gamma }E_{K\text{0}}+\text{5}\text{5}}{\text{8}\text{0}}),\\ \text{0}.\text{1}\text{2}\text{5}\text{exp}(-\frac{V+E_{K\text{0}}+\text{5}\text{5}}{\text{8}\text{0}}),\varepsilon_{m}\geq \varepsilon_{T} \end{array}\right.\; \end{array} \end{array}$$ (17)

The form of Equation (17) also indicates that the gating rates of Nav and Kv channels both undergo a certain injury-induced shift after deformation. For Nav channels, since *E*_*Na0*_ is positive, the model inherently incorporates the Nav left-shift mechanism reported by Wang et al. (2009).

### 4.4. Improved coupling method

Although Jérusalem et al. (2014). implemented damage coupling from the perspective of axonal membrane deformation through Equations (15)–(17), to directly establish a connection between deformation and electrophysiological parameters, we still perform the coupling directly from the perspective of micro-axon strain as shown in Equation (3). For an axon *i* with a strain of $\epsilon_{ma}^{i}$, its damage parameter *ω*^*i*^_*D*_ is defined as,


$$\begin{array}{c} {\omega^{i}}_{D}=(\frac{{\varepsilon^{i}}_{ma}}{{\varepsilon^{i}}_{T}}{)}^{\gamma^{i}} \end{array}$$ (18)

Since the mechanical injury-induced cytoskeletal disruption or axoplasmic transport impairment can lead to varying degrees of focal axon swelling following Ranvier nodes damage [58–60], where ion channels may exist in both injured and uninjured states simultaneously. To simplify the analysis, we adopted the approach of Boucher et al. [30] and divided the Nav and Kv currents in Equation (7) into injured and uninjured phases in proportion to *ω*^*i*^_*D*_,


$$\begin{array}{c} \begin{array}{c} I_{Na}^{i}=\left\{ \begin{array}{c} {\overset{―}{g}}_{Na}[{m^{i}}^{\text{3}}h^{i}(V^{i}-{E^{i}}_{Na\text{0}})(\text{1}-{\omega^{i}}_{D})+{{m^{i}}_{D}}^{\text{3}}{h^{i}}_{D}(V^{i}-{E^{i}}_{Na}){\omega^{i}}_{D}],{\omega^{i}}_{D}<\text{1}\\ {\overset{―}{g}}_{Na}{{m^{i}}_{D}}^{\text{3}}{h^{i}}_{D}\left(V^{i}-{E^{i}}_{Na}\right),{\omega^{i}}_{D}\geq \text{1} \end{array}\right.\;\\ I_{K}^{i}=\left\{ \begin{array}{c} {\overset{―}{g}}_{K}\left[{n^{i}}^{\text{4}}\left(V^{i}-{E^{i}}_{K\text{0}}\right)\left(\text{1}-{\omega^{i}}_{D}\right)+{{n^{i}}_{D}}^{\text{4}}\left(V^{i}-{E^{i}}_{K}\right){\omega^{i}}_{D}\right],{\omega^{i}}_{D}<\text{1}\\ {\overset{―}{g}}_{K}{{n^{i}}_{D}}^{\text{4}}\left(V^{i}-{E^{i}}_{K}\right),{\omega^{i}}_{D}\geq \text{1} \end{array}\right.\; \end{array} \end{array}$$ (19)

Where all damaged components are denoted by the subscript *D*. When *ω*^*i*^_*D*_ < 1, the axon is in an incomplete damage state; when *ω*^*i*^_*D*_ ≥ 1, it indicates that all ion channels at the node have been damaged. In Equation (19), the damaged $E_{Na}^{i}$ and $E_{K}^{i}$ both follow the form of Equation (16), namely $E_{Na}^{i}=E_{Na\text{0}}^{i}(\text{1}-{\omega^{i}}_{D})$ and $E_{K}^{i}=E_{K\text{0}}^{i}(\text{1}-{\omega^{i}}_{D})$. When *ω*^*i*^_*D*_ ≥ 1, both $E_{Na}^{i}$ and $E_{K}^{i}$ reduce to 0 mV. The gating variables and rate constants of the damaged channels are also rewritten in the following form,


$$\begin{array}{c} \frac{d{x^{i}}_{D}}{dt}={\alpha^{i}}_{x,D}(\text{1}-{x^{i}}_{D})-{\beta^{i}}_{x,D}{x^{i}}_{D},\begin{array}{cc} & x\to m,h,n \end{array} \end{array}$$ (20)

$$\begin{array}{c} \begin{array}{c} \alpha_{m,D}^{i}=\left\{ \begin{array}{c} \text{0}.\text{1}\frac{\left(V^{i}+{\omega^{i}}_{D}E_{Na}^{eq}+\text{40}\right)}{\text{1}-\text{exp}(-\frac{V^{i}+{\omega^{i}}_{D}E_{Na}^{eq}+\text{40}}{\text{10}})},\\ \text{0}.\text{1}\frac{\left(V^{i}+E_{Na}^{eq}+\text{40}\right)}{\text{1}-\text{exp}(-\frac{V^{i}+E_{Na}^{eq}+\text{40}}{\text{10}})},{\omega^{i}}_{D}\geq \text{1} \end{array}\right.\;\\ \beta_{m,D}^{i}=\left\{ \begin{array}{c} \text{4exp}(-\frac{V^{i}+{\omega^{i}}_{D}E_{Na}^{eq}+\text{65}}{\text{18}}),\\ \text{4exp}(-\frac{V^{i}+E_{Na}^{eq}+\text{65}}{\text{18}}),{\omega^{i}}_{D}\geq \text{1} \end{array}\right.\;\\ \alpha_{h,D}^{i}=\left\{ \begin{array}{c} \text{0}.\text{07exp}(-\frac{V^{i}+{\omega^{i}}_{D}E_{Na}^{eq}+\text{65}}{\text{20}}),\\ \text{0}.\text{07exp}(-\frac{V^{i}+E_{Na}^{eq}+\text{65}}{\text{20}}),{\omega^{i}}_{D}\geq \text{1} \end{array}\right.\;\\ \beta_{h,D}^{i}=\left\{ \begin{array}{c} \frac{\text{1}}{\text{1}+\text{exp}(-\frac{V^{i}+{\omega^{i}}_{D}E_{Na}^{eq}+\text{35}}{\text{10}})},\\ \frac{\text{1}}{\text{1}+\text{exp}(-\frac{V^{i}+E_{Na}^{eq}+\text{35}}{\text{10}})},{\omega^{i}}_{D}\geq \text{1} \end{array}\right.\;\\ \alpha_{n,D}^{i}=\left\{ \begin{array}{c} \text{0}.\text{01}\frac{\left(V^{i}+{\omega^{i}}_{D}E_{K}^{eq}+\text{55}\right)}{\text{1}-\text{exp}(-\frac{V^{i}+{\omega^{i}}_{D}E_{K}^{eq}+\text{55}}{\text{10}})},\\ \text{0}.\text{01}\frac{\left(V^{i}+E_{K}^{eq}+\text{55}\right)}{\text{1}-\text{exp}(-\frac{V^{i}+E_{K}^{eq}+\text{55}}{\text{10}})},{\omega^{i}}_{D}\geq \text{1} \end{array}\right.\;\\ \beta_{n,D}^{i}=\left\{ \begin{array}{c} \text{0}.\text{125exp}(-\frac{V^{i}+{\omega^{i}}_{D}E_{K}^{eq}+\text{55}}{\text{80}}),\\ \text{0}.\text{125exp}(-\frac{V^{i}+E_{K}^{eq}+\text{55}}{\text{80}}),{\omega^{i}}_{D}\geq \text{1} \end{array}\right.\; \end{array} \end{array}$$ (21)

For the gating rate constants of damaged channels in Equation (20), we do not adopt the non-interfering form shown in Equation (17); instead, we comprehensively consider the coupled effects of Nav and Kv channels. This is because the direct or indirect influences of functional changes in Nav and Kv channels on each other have been studied in contexts such as certain neurological diseases (including Brugada syndrome, spinocerebellar ataxia, and demyelinating diseases like multiple sclerosis) as well as in the homeostatic regulation of neuronal excitability [61–63]. Moreover, due to the difference in the spatial distribution of Nav and Kv ion channels at the Ranvier nodes—Kv channels are mainly located in the adjacent myelin regions, while Nav channels are concentrated in the unmyelinated areas [56,64]—the translation value characterizing their damage may not be the same as that of Nav channels under the same deformation conditions. Therefore, $E_{Na}^{eq}$ and $E_{K}^{eq}$ in Equation (21) are written in the form of *E*_*Na0*_ and *E*_*K0*_ acting together,


$$\begin{array}{c} \begin{array}{c} E_{Na}^{eq}=(\text{1}-C_{Na})E_{K\text{0}}+C_{Na}E_{Na\text{0}}\\ E_{K}^{eq}=(\text{1}-C_{K})E_{Na\text{0}}+C_{K}E_{K\text{0}}\\ C_{Na}=C_{K}=C \end{array} \end{array}$$ (22)

Where *C*_*Na*_ and *C*_*K*_ are the respective translation coefficients of Nav and Kv channels. To satisfy the condition of Nav left-shift, while avoiding excessively strong spontaneous spiking and an overly negative resting potential, after substituting the electrophysiological parameter values listed in Table 3, *C*_*Na*_ in Equation (22) needs to be greater than 0.7, and *C*_*K*_ needs to be greater than 0.65. Herein, *C*_*Na*_ and *C*_*K*_ are represented by a single unknown parameter *C*. On the one hand, they satisfy highly similar constraint conditions, which mathematically justifies unifying them into one identical parameter. On the other hand, as stated above, recent studies have confirmed direct molecular‑level interactions between Nav and Kv channels. For instance, they can assemble into “channelosome” complexes via auxiliary subunits (e.g., Na_v_β1) to mutually modulate gating and membrane trafficking [61–63]. Given the current lack of experimental data that can quantitatively distinguish the respective damage shift magnitudes of Nav and Kv channels, adopting a single parameter *C* guarantees parameter identifiability as well as model parsimony.

### 4.5. Statistical damage coupling at the nerve bundle level

For a single axon, when its microscopic axonal strain *ε_ma_* is determined, the corresponding damage parameter *ω*_*D*_ is also uniquely determined. However, due to the heterogeneity of the tissue structure in the actual nerve bundle and the differences in the initial morphological properties among individual axons, under the same macroscopic deformation loading (as shown in Equation (18)), the microscopic axonal strain *ε_ma_*, disability strain threshold *ε*_*T*_, and parameter *γ* of each axon may vary, leading to differences in their respective damage parameters *ω*_*D*_. To simplify the analysis, as shown in Fig 11(a), we assume that the damage parameter *ω*_*D*_ of each axon follows a specific probability density distribution *f(ω*_*D*_*)*, with a mean value of *M*—indicating that the values of *ω*_*D*_ are concentrated around *M*. The probability density function is selected in the form of the Weibull distribution, as follows,

**Fig 11:**
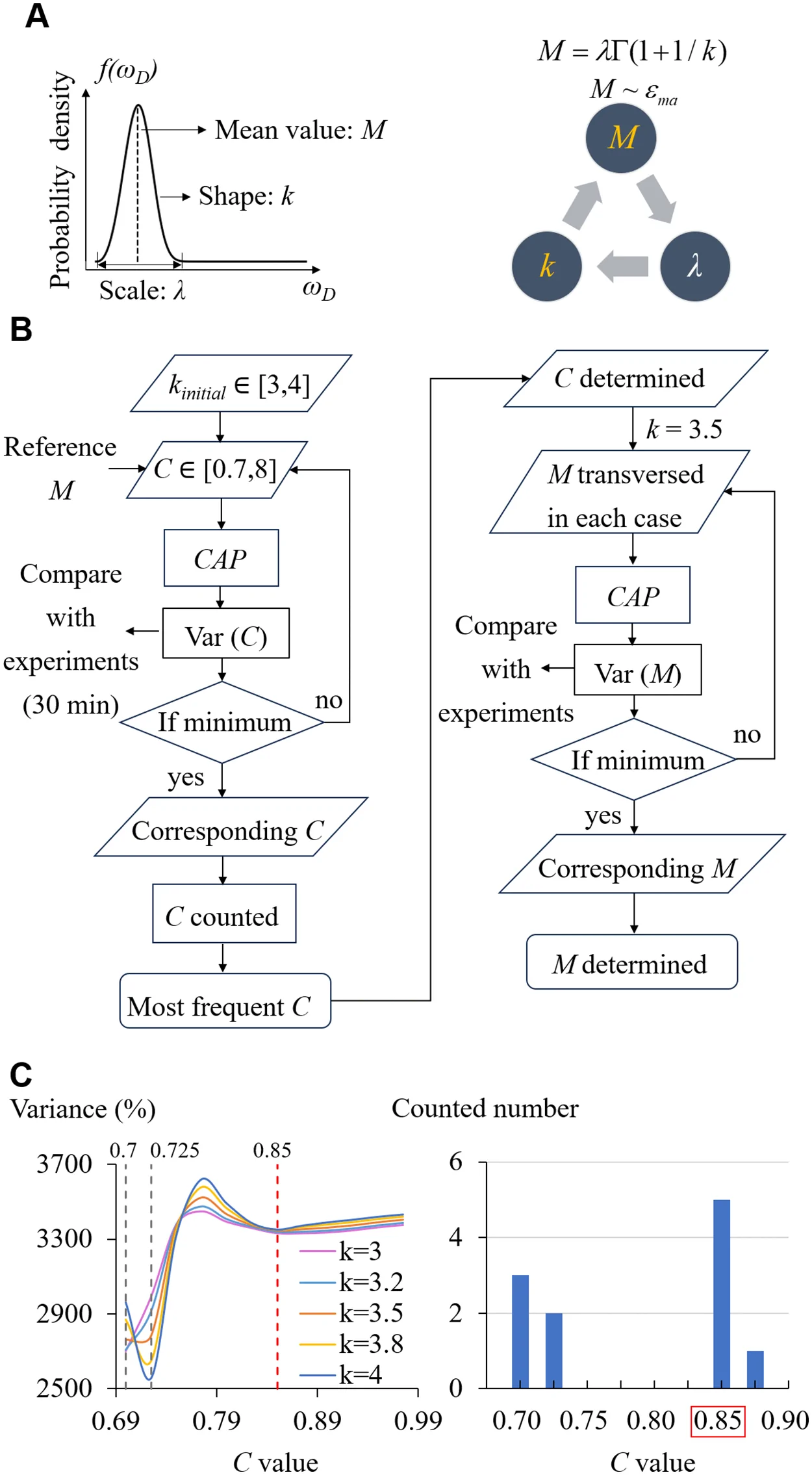
Parameter definition and iterative determination method of the damage model. The parameters include *C*, *k*, and the mean value *M* under each working condition. **(a)** Parameter interpretation of the Weibull distribution: The shape parameter *k* controls the shape and variation trend of the probability density function (pdf) curve, while the scale parameter *λ* controls the distribution range and position of the pdf curve. Please note that the final determined parameters of this paper will cause the pdf curve to exhibit a slight positive skewness. Both parameters have a mapping relationship with the distribution mean *M*; therefore, only two of these parameters need to be determined to clarify the overall distribution characteristics. In this paper, *k* and *M* are selected for determination (as shown in the highlighted part). **(b)** The full process of directly determining model parameters from the nerve bundle level. Within the given initial value range of *k*, the value of *C* is first determined based on the results of the experiment conducted by Shi and Whitebone (2006) at 30 minutes, and then *k* = 3.5 is used to determine the values of *M* under each working condition at the remaining time points. **(c)** Schematic diagram for determining the value of ***C*.** The left side shows the variation of the variance curve with *C* under different initial values of k; the right side shows the number of occurrences of *C* corresponding to the minimum variance, where the corresponding *C* values are marked by dashed lines. Among these, *C* = 0.85 has the highest count, so it is marked with a red dashed line and a red frame.


$$\begin{array}{c} f(\omega_{D})=\frac{k}{\lambda }(\frac{\omega_{D}}{\lambda }{)}^{k-\text{1}}\text{exp}(-(\frac{\omega_{D}}{\lambda }{)}^{k}) \end{array}$$ (23)

Where *k* is the shape parameter, which is used to regulate the morphology of the probability density curve; *λ* is the scale parameter, which is used to control the range and position of the distribution. When the number of microscopic axons in the nerve bundle is sufficiently large, the proportion of axons with the damage parameter *ω*_*D*_ can be approximately expressed as *f(ω*_*D*_*)d*ω_*D*_. Under the assumption that the leakage current and applied current are uniformly distributed across the macroscopic white matter bundle at the same cross-section, combined with Equations (19), and (23), the Nav and Kv currents of all axons are summed to obtain the total membrane potential equation at the macroscopic level, as follows,


$$\begin{array}{c} {C_{M}\frac{dV}{dt}|}_{total}=-g_{l}(V-E_{l})+I_{ext}-\int_{\text{0}}^{\infty }I_{Na}f(\omega_{D})d\omega_{D}-\int_{\text{0}}^{\infty }I_{K}f(\omega_{D})d\omega_{D} \end{array}$$ (24)

For the Weibull distribution, there is the following relationship between the shape parameter *k*, the scale parameter *λ*, and the distribution mean *M*,


$$\begin{array}{c} M=\lambda \Gamma (\text{1}+\text{1}/k) \end{array}$$ (25)

Where *Γ(·)* is gamma function. Hence, for the Weibull distribution, the entire distribution curve can be uniquely determined by specifying any two of the three parameters: *k*, *λ*, and *M*, as shown in Fig 11(b). For the same type of nerve bundles, we propose that the morphology of the damage distribution curve should be consistent under the same loading type, given the overall consistency of their internal strength. Thus, we set *k* as an undetermined coefficient. Since *M* is the mean value of the damage distribution, which can reflect the overall damage level and is directly related to axonal deformation, it is defined as an undetermined coefficient associated with axonal strain. All other electrophysiological parameters are detailed in Table 3.

It should be noted that the adopted Weibull distribution exhibits a slight positive skewness rather than perfect symmetry. Such skewed behavior is consistent with experimental measurements of micro‑axonal diameter distributions within nerve fibers [65–67]. As for setting the upper integral limit in Equation (24) to infinity, this treatment stems from two considerations. First, the probability‑density function of the Weibull distribution is intrinsically defined over the domain [0,+∞). Second, owing to the large population of micro‑axons, direct discrete summation is computationally intractable, and the strain values of individual axons may span a broad range. As reported in [15], even under modest macroscopic deformation, the strain at the axonal node of Ranvier can be substantially higher than the bulk strain of the axon. Nevertheless, the distribution function prevents an over‑abundant fraction of axons from attaining extremely large strain values.

### 4.6. Determination of statistical damage model parameters

Based on the modeling process described above, three core model parameters need to be determined ultimately: the Nav and Kv channel translation coefficient *C*, the shape parameter *k* of the damage distribution, and the distribution mean *M*. The following sections will describe the procedure to determine these parameters.

#### 4.6.1. Reference experimental loading conditions.

As illustrated before, we referred to the classic spinal cord injury experiment by Shi and Whitebone (2006) to calibrate model parameters. Specifically, the spinal strips were stretched to three different strain levels (0.25, 0.5, and 1.0, corresponding to “mild”, “moderate”, and “severe”) under the strain rates of “fast” and “slow”(355–519 s^-1^ and 0.006-0.008 s^-1^, respectively), and then the load was removed for free relaxation. Within 0–30 minutes after injury, the samples were stimulated with pulsed current every 5 minutes, and the CAP recovery level was measured. A total of 6 working conditions and 7 measurement time points were conducted, with the specific working condition parameters detailed in Table 1.

#### 4.6.2. Simulation settings.

After calculating axonal strains under different loading conditions and time points via Equations (3)–(6) using parameters listed in Table 1, we performed electrophysiological simulations based on the injury coupling model described earlier. Simulations were implemented in Python 3.11 with the Brian2 2.7 simulator [68], using the fourth-order Runge-Kutta method for numerical solution. Each simulation lasted 120 ms, with a pulse current of 50 μA/cm² applied at 50 ms to elicit compound action potentials (CAPs). The definitions of CAP amplitude and recovery level are as follows,


$$\begin{array}{c} CAP=V_{spike}-V_{rest} \end{array}$$ (26)

$$\begin{array}{c} Amplitude(\%)=\frac{CAP_{injured}}{CAP_{normal}} \end{array}$$ (27)

Where *V*_*spike*_ is the spiking potential, *V*_*rest*_ is the resting potential. *V*_*spike*_ is derived from the peak value of the membrane potential activated after stimulation, while *V*_*rest*_ is derived from the average value of the membrane potential in the 20 ms before the end of the simulation, after the membrane potential has returned to a stable state. All other simulation parameters are shown in Table 3.

#### 4.6.3. Iteration steps for solving model parameters to simulate experiments.

As shown in Fig 11(b), we determine the model parameters directly from the quantity level of nerve bundles. Under the assumption that the damage variable *ω*_*D*_ should be concentrated around the mean value *M*, the shape parameter *k* is set within the range of 3–4. Therefore, different initial values of *k* are selected within this range for the nonlinear fitting of *C*. The fitting is based on calculating the corresponding CAP values under a given reference mean *M*, as well as the variance between these CAP values and the results at 30 minutes in the experiment by Shi and Whitebone (2006). The reference mean *M* for each working condition is set in the form expressed by *ε*_*m*_ and *ε*_*T*_ in the work of Jérusalem et al. When the variance reaches a minimum value with the traversed *C* values, the corresponding *C* values are counted, and finally, the *C* value with the highest count is taken as the value determined by fitting. Fig 11(c) shows a schematic diagram for determining the *C* value; it can be seen that under different initial values of k, the variance curves all reach their minimum values around *C* = 0.85, and this *C* value also has the highest count. Therefore, it is selected as the finally determined *C* value.

Since *k* converges to the same *C* value within the given interval, after determining the *C* value, *k* = 3.5 is directly adopted to determine the distribution mean *M* corresponding to each working condition. The process is similar to the aforementioned method for determining the *C* value, but the fitting range at this point includes the results at all time points in Shi and Whitebone’s experiment.

To verify the numerical stability of the model, we investigated the influences of initial conditions, together with parameters *k* and *C*, on membrane potential traces and CAP outputs. Meanwhile, a convergence analysis of *dt* and *dω*_*D*_ in Equation (24) was conducted to verify the solution robustness of the model. In addition, to verify the reliability of the model outputs, Monte‑Carlo‑based uncertainty analysis was performed for each loading case at the 30 min time point as an example. Detailed results are presented individually in Fig A, Fig B and Fig C in S1 Appendix. The results show that the output consistency, numerical stability, and the reliability of damage prediction of our model can be guaranteed.

### 4.7. Code accessibility

All custom codes supporting the theoretical model and simulations in this paper are available in Supplemental Materials.

## Supporting information

S1 Code Supplementary codes. A.zip file of the Python codes used for axonal stretch simulation, model parameter determining, compound action potential calculation, and plots for Weibull distribution curves. The codes used for sensitivity, convergence and uncertainty analysis of the model are also included.
(ZIP)

S1 Data Supplementary data. An.xlsx file presenting all data illustrated in Fig 2(c), Figs 3–7, and Fig 11.
(XLSX)

S1 Text Readme. A.txt file for illustrating how to run the simulation codes. (TXT)

S1 Appendix Supplementary analysis. A.docx file describing our parameter sensitivity, convergence and uncertainty analysis of the proposed model. Fig A. A.tif file showing the results of the sensitivity analysis. Fig B. A.tif file showing the results of the convergence analysis. Fig C. A.tif file showing the results of the uncertainty analysis.
(DOCX)

## References

[1] Gennarelli TA, Thibault LE, Adams JH, Graham DI, Thompson CJ, Marcincin RP. Diffuse axonal injury and traumatic coma in the primate. Ann Neurol. 1982;12(6):564–74. doi: 10.1002/ana.410120611 7159060

[2] Bain AC, Meaney DF. Tissue-level thresholds for axonal damage in an experimental model of central nervous system white matter injury. J Biomech Eng. 2000;122(6):615–22. doi: 10.1115/1.1324667 11192383

[3] Oberholzer M, Müri RM. Neurorehabilitation of traumatic brain injury (TBI): A clinical review. Med Sci (Basel). 2019;7(3):47. doi: 10.3390/medsci7030047 30889900 PMC6473767

[4] Sullivan D, Vaglio BJ, Cararo-Lopes MM, Wong RDP, Graudejus O, Firestein BL. Stretch-induced injury affects cortical neuronal networks in a time- and severity-dependent manner. Ann Biomed Eng. 2024;52:1021–38. doi: 10.1007/s10439-023-03438-0 38294641

[5] Shi R, Whitebone J. Conduction deficits and membrane disruption of spinal cord axons as a function of magnitude and rate of strain. J Neurophysiol. 2006;95(6):3384–90. doi: 10.1152/jn.00350.2005 16510778

[6] Zarmer L, Khan M, Islat G, Alameddin H, Massey M, Chaudhry R. Traumatic spinal cord injury: Review of the literature. J Clin Med. 2025;14(11):3649. doi: 10.3390/jcm14113649 40507410 PMC12155255

[7] Meythaler JM, Peduzzi JD, Eleftheriou E, Novack TA. Current concepts: Diffuse axonal injury-associated traumatic brain injury. Arch Phys Med Rehabil. 2001;82(10):1461–71. doi: 10.1053/apmr.2001.25137 11588754

[8] Blight A. Mechanical factors in experimental spinal cord injury. J Am Paraplegia Soc. 1988;11(2):26–34. doi: 10.1080/01952307.1988.11735792 3076595

[9] Shi R, Borgens RB. Acute repair of crushed guinea pig spinal cord by polyethylene glycol. J Neurophysiol. 1999;81(5):2406–14. doi: 10.1152/jn.1999.81.5.2406 10322076

[10] Shi R, Pryor JD. Pathological changes of isolated spinal cord axons in response to mechanical stretch. Neuroscience. 2002;110(4):765–77. doi: 10.1016/s0306-4522(01)00596-6 11934483

[11] Ellis EF, McKinney JS, Willoughby KA, Liang S, Povlishock JT. A new model for rapid stretch-induced injury of cells in culture: Characterization of the model using astrocytes. J Neurotrauma. 1995;12(3):325–39. doi: 10.1089/neu.1995.12.325 7473807

[12] Slemmer JE, Matser EJT, De Zeeuw CI, Weber JT. Repeated mild injury causes cumulative damage to hippocampal cells. Brain. 2002;125(Pt 12):2699–709. doi: 10.1093/brain/awf271 12429597

[13] Yap YC, Dickson TC, King AE, Breadmore MC, Guijt RM. Microfluidic culture platform for studying neuronal response to mild to very mild axonal stretch injury. Biomicrofluidics. 2014;8(4):044110. doi: 10.1063/1.4891098 25379095 PMC4189213

[14] Yap YC, King AE, Guijt RM, Jiang T, Blizzard CA, Breadmore MC, et al. Mild and repetitive very mild axonal stretch injury triggers cystoskeletal mislocalization and growth cone collapse. PLoS One. 2017;12(5):e0176997. doi: 10.1371/journal.pone.0176997 28472086 PMC5417565

[15] Zhu F, Gatti DL, Yang KH. Nodal versus total axonal strain and the role of cholesterol in traumatic brain injury. J Neurotrauma. 2016;33(9):859–70. doi: 10.1089/neu.2015.4007 26393780

[16] Montanino A, Kleiven S. Utilizing a structural mechanics approach to assess the primary effects of injury loads onto the axon and its components. Front Neurol. 2018;9:643. doi: 10.3389/fneur.2018.00643 PMC6087765 30127763

[17] Zhang C, Ji S. Sex differences in axonal dynamic responses under realistic tension using finite element models. J Neurotrauma. 2023;40(19–20):2217–32. doi: 10.1089/neu.2022.0512 37335051

[18] Galbraith JA, Thibault LE, Matteson DR. Mechanical and electrical responses of the squid giant axon to simple elongation. J Biomech Eng. 1993;115(1):13–22. doi: 10.1115/1.2895464 8445893

[19] Bain AC, Raghupathi R, Meaney DF. Dynamic stretch correlates to both morphological abnormalities and electrophysiological impairment in a model of traumatic axonal injury. J Neurotrauma. 2001;18(5):499–511. doi: 10.1089/089771501300227305 11393253

[20] LaPlaca MC, Simon CM, Prado GR, Cullen DK. CNS injury biomechanics and experimental models. Prog Brain Res. 2007;161:13–26. doi: 10.1016/S0079-6123(06)61002-9 17618967

[21] Singh A, Kallakuri S, Chen C, Cavanaugh JM. Structural and functional changes in nerve roots due to tension at various strains and strain rates: An in-vivo study. J Neurotrauma. 2009;26(4):627–40. doi: 10.1089/neu.2008.0621 19271962

[22] Rickett T, Connell S, Bastijanic J, Hegde S, Shi R. Functional and mechanical evaluation of nerve stretch injury. J Med Syst. 2011;35:787–93. doi: 10.1007/s10916-010-9468-1 20703726

[23] Maxwell WL, Povlishock JT, Graham DL. A mechanistic analysis of nondisruptive axonal injury: A review. J Neurotrauma. 1997;14(7):419–40. doi: 10.1089/neu.1997.14.419 9257661

[24] Wolf JA, Stys PK, Lusardi T, Meaney D, Smith DH. Traumatic axonal injury induces calcium influx modulated by tetrodotoxin-sensitive sodium channels. J Neurosci. 2001;21(6):1923–30. doi: 10.1523/JNEUROSCI.21-06-01923.2001 11245677 PMC6762603

[25] Iwata A, Stys PK, Wolf JA, Chen X-H, Taylor AG, Meaney DF, et al. Traumatic axonal injury induces proteolytic cleavage of the voltage-gated sodium channels modulated by tetrodotoxin and protease inhibitors. J Neurosci. 2004;24(19):4605–13. doi: 10.1523/JNEUROSCI.0515-03.2004 15140932 PMC6729402

[26] Shcherbatko A, Ono F, Mandel G, Brehm P. Voltage-dependent sodium channel function is regulated through membrane mechanics. Biophysical Journal. 1999;77:1945–59. doi: 10.1016/S0006-3495(99)77036-0 PMC1300476 10512815

[27] Tabarean IV, Juranka P, Morris CE. Membrane stretch affects gating modes of a skeletal muscle sodium channel. Biophys J. 1999;77(2):758–74. doi: 10.1016/S0006-3495(99)76930-4 10423424 PMC1300370

[28] Morris CE, Juranka PF. Nav channel mechanosensitivity: Activation and inactivation accelerate reversibly with stretch. Biophys J. 2007;93(3):822–33. doi: 10.1529/biophysj.106.101246 17496023 PMC1913161

[29] Wang JA, Lin W, Morris T, Banderali U, Juranka PF, Morris CE. Membrane trauma and Na leak from Nav1.6 channels. American Journal of Physiology-Cell Physiology. 2009;297:C823–34. doi: 10.1152/ajpcell.00505.2008 19657055

[30] Boucher P-A, Joós B, Morris CE. Coupled left-shift of Nav channels: Modeling the Na⁺-loading and dysfunctional excitability of damaged axons. J Comput Neurosci. 2012;33(2):301–19. doi: 10.1007/s10827-012-0387-7 22476614

[31] Jérusalem A, García-Grajales JA, Merchán-Pérez A, Peña JM. A computational model coupling mechanics and electrophysiology in spinal cord injury. Biomech Model Mechanobiol. 2014;13(4):883–96. doi: 10.1007/s10237-013-0543-7 24337934

[32] Cinelli I, Destrade M, McHugh P, Trotta A, Gilchrist M, Duffy M. Head-to-nerve analysis of electromechanical impairments of diffuse axonal injury. Biomech Model Mechanobiol. 2019;18(2):361–74. doi: 10.1007/s10237-018-1086-8 30430371

[33] Garcia-Gonzalez D, Jerusalem A. Energy based mechano-electrophysiological model of CNS damage at the tissue scale. Journal of the Mechanics and Physics of Solids. 2019;125:22–37. doi: 10.1016/j.jmps.2018.12.009

[34] Tian J, Huang G, Lin M, Qiu J, Sha B, Lu TJ, et al. A mechanoelectrical coupling model of neurons under stretching. J Mech Behav Biomed Mater. 2019;93:213–21. doi: 10.1016/j.jmbbm.2019.02.007 30826698

[35] Silva NRFA, Bonfante EA, Zavanelli RA, Thompson VP, Ferencz JL, Coelho PG. Reliability of metalloceramic and zirconia-based ceramic crowns. J Dent Res. 2010;89(10):1051–6. doi: 10.1177/0022034510375826 20660796 PMC3318053

[36] Joshi A, Haththotuwa N, Richard JS, Laven R, Dias GJ, Staiger MP. In vitro calibration and in vivo validation of phenomenological corrosion models for resorbable magnesium-based orthopaedic implants. Acta Biomater. 2024;180:171–82. doi: 10.1016/j.actbio.2024.03.024 38570108

[37] Hajizadeh P, Khosravi M, Ravandi M. Failure analysis of advanced ceramics using bivariate Weibull distribution and Bayesian estimation. Engineering with Computers. 2025;41(5):2989–3003. doi: 10.1007/s00366-025-02143-x

[38] Wang J, Geng H, Li P. A software reliability model for open source big data systems based on Weibull-Weibull distribution. Sci Rep. 2025;15(1):14670. doi: 10.1038/s41598-025-98942-9 40287525 PMC12033355

[39] Beyder A, Rae JL, Bernard C, Strege PR, Sachs F, Farrugia G. Mechanosensitivity of Nav1.5, a voltage-sensitive sodium channel. J Physiol. 2010;588(Pt 24):4969–85. doi: 10.1113/jphysiol.2010.199034 21041530 PMC3036191

[40] Ding M-C, Wang Q, Lo EH, Stanley GB. Cortical excitation and inhibition following focal traumatic brain injury. J Neurosci. 2011;31(40):14085–94. doi: 10.1523/JNEUROSCI.3572-11.2011 21976493 PMC3444158

[41] Marsh B, Chauvette S, Huang M, Timofeev I, Bazhenov M. Network effects of traumatic brain injury: From infra slow to high frequency oscillations and seizures. J Comput Neurosci. 2025;53(2):247–66. doi: 10.1007/s10827-025-00895-5 40019646 PMC12181067

[42] González OC, Krishnan GP, Timofeev I, Bazhenov M. Ionic and synaptic mechanisms of seizure generation and epileptogenesis. Neurobiol Dis. 2019;130:104485. doi: 10.1016/j.nbd.2019.104485 31150792 PMC6689444

[43] Yu N, Morris CE, Joós B, Longtin A. Spontaneous excitation patterns computed for axons with injury-like impairments of sodium channels and Na/K pumps. PLoS Comput Biol. 2012;8(9):e1002664. doi: 10.1371/journal.pcbi.1002664 23028273 PMC3441427

[44] Zhang Y, Yang D, Fan D, Wang H, Chen Y, Chen Y. Unraveling the dynamics of firing patterns for neurons with impairment of sodium channels. Chaos. 2024;34(10):103132. doi: 10.1063/5.0223512 39413258

[45] Devor M, Wall PD. Cross-excitation in dorsal root ganglia of nerve-injured and intact rats. J Neurophysiol. 1990;64(6):1733–46. doi: 10.1152/jn.1990.64.6.1733 2074461

[46] Liu C-N, Devor M, Waxman SG, Kocsis JD. Subthreshold oscillations induced by spinal nerve injury in dissociated muscle and cutaneous afferents of mouse DRG. J Neurophysiol. 2002;87(4):2009–17. doi: 10.1152/jn.00705.2001 11929919 PMC2613787

[47] Zhang X-F, Zhu CZ, Thimmapaya R, Choi WS, Honore P, Scott VE, et al. Differential action potentials and firing patterns in injured and uninjured small dorsal root ganglion neurons after nerve injury. Brain Res. 2004;1009(1–2):147–58. doi: 10.1016/j.brainres.2004.02.057 15120592

[48] Idlett-Ali S, Kloefkorn H, Goolsby W, Hochman S. Relating spinal injury-induced neuropathic pain and spontaneous afferent activity to sleep and respiratory dysfunction. Journal of Neurotrauma. 2023;40:2654–66. doi: 10.1089/neu.2022.0305 PMC11093096 37212274

[49] Mao Q, Yuan J, Ming X, Wu S, Chen L, Bekker A, et al. Role of dorsal root ganglion K2p1.1 in peripheral nerve injury-induced neuropathic pain. Mol Pain. 2017;13:1744806917701135. doi: 10.1177/1744806917701135 28326939 PMC5367768

[50] Zhang J, Rong L, Shao J, Zhang Y, Liu Y, Zhao S, et al. Epigenetic restoration of voltage-gated potassium channel Kv1.2 alleviates nerve injury-induced neuropathic pain. J Neurochem. 2021;156(3):367–78. doi: 10.1111/jnc.15117 32621322

[51] Sunderland S. The anatomy and physiology of nerve injury. Muscle Nerve. 1990;13(9):771–84. doi: 10.1002/mus.880130903 2233864

[52] Pfister BJ, Iwata A, Meaney DF, Smith DH. Extreme stretch growth of integrated axons. J Neurosci. 2004;24(36):7978–83. doi: 10.1523/JNEUROSCI.1974-04.2004 15356212 PMC6729931

[53] Sousa SC, Aroso M, Bessa R, Veríssimo E, Ferreira da Silva T, Lopes CDF, et al. Stretch triggers microtubule stabilization and MARCKS-dependent membrane incorporation in the shaft of embryonic axons. Curr Biol. 2024;34(19):4577-4588.e8. doi: 10.1016/j.cub.2024.08.018 39265571

[54] Bonakdar N, Gerum R, Kuhn M, Spörrer M, Lippert A, Schneider W, et al. Mechanical plasticity of cells. Nat Mater. 2016;15(10):1090–4. doi: 10.1038/nmat4689 27376682

[55] Hodgkin AL, Huxley AF. A quantitative description of membrane current and its application to conduction and excitation in nerve. J Physiol. 1952;117(4):500–44. doi: 10.1113/jphysiol.1952.sp004764 12991237 PMC1392413

[56] Nashmi R, Fehlings MG. Mechanisms of axonal dysfunction after spinal cord injury: with an emphasis on the role of voltage-gated potassium channels. Brain Res Brain Res Rev. 2001;38(1–2):165–91. doi: 10.1016/s0165-0173(01)00134-5 11750932

[57] Ouyang H, Sun W, Fu Y, Li J, Cheng J-X, Nauman E, et al. Compression induces acute demyelination and potassium channel exposure in spinal cord. J Neurotrauma. 2010;27(6):1109–20. doi: 10.1089/neu.2010.1271 20373847 PMC2943505

[58] Tang-Schomer MD, Patel AR, Baas PW, Smith DH. Mechanical breaking of microtubules in axons during dynamic stretch injury underlies delayed elasticity, microtubule disassembly, and axon degeneration. FASEB J. 2010;24(5):1401–10. doi: 10.1096/fj.09-142844 20019243 PMC2879950

[59] Wu Y-T, Gilpin K, Adnan A. Effects of focal axonal swelling level on the action potential signal transmission. J Comput Neurosci. 2020;48(3):253–63. doi: 10.1007/s10827-020-00750-9 32436129

[60] Pan X, Li J, Li W, Wang H, Durisic N, Li Z, et al. Axons-on-a-chip for mimicking non-disruptive diffuse axonal injury underlying traumatic brain injury. Lab Chip. 2022;22(23):4541–55. doi: 10.1039/d2lc00730d 36318066

[61] Bittner S, Meuth SG. Targeting ion channels for the treatment of autoimmune neuroinflammation. Ther Adv Neurol Disord. 2013;6(5):322–36. doi: 10.1177/1756285613487782 23997817 PMC3755531

[62] Clatot J, Neyroud N, Cox R, Souil C, Huang J, Guicheney P, et al. Inter-regulation of kv4.3 and voltage-gated sodium channels underlies predisposition to cardiac and neuronal channelopathies. Int J Mol Sci. 2020;21(14):5057. doi: 10.3390/ijms21145057 32709127 PMC7404392

[63] Li B, Suutari BS, Sun SD, Luo Z, Wei C, Chenouard N, et al. Neuronal inactivity co-opts LTP machinery to drive potassium channel splicing and homeostatic spike widening. Cell. 2020;181(7):1547-1565.e15. doi: 10.1016/j.cell.2020.05.013 32492405 PMC9310388

[64] Volman V, Ng LJ. Primary paranode demyelination modulates slowly developing axonal depolarization in a model of axonal injury. J Comput Neurosci. 2014;37(3):439–57. doi: 10.1007/s10827-014-0515-7 24986633

[65] Tomasi S, Caminiti R, Innocenti GM. Areal differences in diameter and length of corticofugal projections. Cereb Cortex. 2012;22(6):1463–72. doi: 10.1093/cercor/bhs011 22302056

[66] Assaf Y, Blumenfeld-Katzir T, Yovel Y, Basser PJ. AxCaliber: A method for measuring axon diameter distribution from diffusion MRI. Magn Reson Med. 2008;59(6):1347–54. doi: 10.1002/mrm.21577 18506799 PMC4667732

[67] Liewald D, Miller R, Logothetis N, Wagner H-J, Schüz A. Distribution of axon diameters in cortical white matter: An electron-microscopic study on three human brains and a macaque. Biol Cybern. 2014;108(5):541–57. doi: 10.1007/s00422-014-0626-2 25142940 PMC4228120

[68] Goodman D, Brette R. Brian: A simulator for spiking neural networks in python. Front Neuroinform. 2008;2:5. doi: 10.3389/neuro.11.005.2008 19115011 PMC2605403

