# Supplementary material for: A statistical damage model for coupled mechano-electrophysiological axonal injury
Source: PLoS Comput Biol. 2026 Oct 9;22(10):e1014837. doi: 10.1371/journal.pcbi.1014837 (PMC13652255; doi:10.1371/journal.pcbi.1014837)
Supplement: Supplementary file 1 [file pcbi.1014837.s004.docx]

**A** **statistical damage model for coupled mechano-electrophysiological axonal injury. S1 Appendix**

Zexuan Chen^1^, Liqun Tang^1*^, Bao Yang^1^, Yiping Liu^1^, Zhenyu Jiang^1^, Licheng Zhou^1^, Zejia Liu^1^

**Introduction：**

This appendix presents descriptions of parameter‑sensitivity analysis, numerical‑convergence analysis, and uncertainty analysis for the coupled nerve‑bundle damage model proposed in the paper *A statistical damage model for coupled mechano‑electrophysiological axonal injury*. The methodologies and corresponding results for each part are presented below. All codes supporting these analyses are accessible in S1 Supplementary codes.

**Sensitivity analysis of initial conditions and model parameters：**

We investigated the influences of initial conditions (*V_0_*, *m_0_*, *h_0_*, and *n_0_*), parameter *C* in Equation (22), and parameter *k* in Equation (23) on membrane potential outputs and CAP amplitudes. The injury case slow‑severe‑30 min was adopted as the example, with the damage distribution mean value *M* kept constant.

For the initial‑condition investigation, *C* = 0.85 and *k* = 3.5 were maintained as in the original manuscript. *V_0_* was randomly sampled within −65mV ± 5mV, while *m_0_*, *h_0_* and *n_0_* were randomly assigned in the range of 0 ~ 1; five individual tests were performed. As shown in Fig A(a) and A(b), although different initial conditions induce considerable deviations in membrane potential curves within the first 20 ms after simulation onset, the curves rapidly converge afterwards. The CAP amplitudes obtained from each test are very close to the reference value of 37.44% reported in the original manuscript.

When examining parameter *C*, the initial conditions were fixed at *V_0_* = −65mV, *m_0_* = *h_0_* = 0, *n_0_* = 0.5, alongside *k* = 3.5, consistent with the original manuscript. Five simulation runs were carried out using a set of values for *C*: 0.7, 0.75, 0.8, 0.85 and 0.9. As shown in Fig A(c), the resulting CAP values remain close to the reference result.

For parameter *k*, the aforementioned initial conditions and *C* = 0.85 were retained. Five simulations were conducted with *k* taking values of 3, 3.5, 4, 4.5 and 5. According to Fig A(d), the output CAP values cluster around the reference amplitude of 37.44%. Collectively, these results demonstrate that the model yields favorable output consistency under varied initial conditions and over a relatively broad parameter range.

**Convergence analysis：**

Considering the complexity of the model, we performed a numerical stability on it. The complexity of the model is ultimately reflected in Equation (24) of the original manuscript：

${C_{M}\frac{dV}{dt}|}_{total}=-g_{l}(V-E_{l})+I_{ext}-\int_{0}^{\infty} I_{Na}f(\omega_{D})d\omega_{D}-\int_{0}^{\infty} I_{K}f(\omega_{D})d\omega_{D}$	(24)

We assign a series of values to the integral steps *dω_D_* and *dt* and compare the membrane potential curves. As illustrated in Fig B, taking the mean value *M* = 1 as an example, with all other parameters kept constant, distinct membrane potential curves gradually converge as *dω_D_* and *dt* decrease, which demonstrates that the model solution tends to be stable when the computational precision is sufficiently high. We adopt the maximum achievable precision within acceptable computational costs, namely *dω_D_* = 1/1000 and *dt* = 0.005 ms.

**Uncertainty analysis：**

Given the probability distribution introduced for the damage parameter *ω_D_*, Monte‑Carlo‑based uncertainty analysis was performed to further examine the reliability of model outputs. All loading cases at the 30 min time point were selected for this analysis. In each simulation, 200 values of *ω_D_* were randomly sampled within the range of 0 ~ 5 as model inputs. This sampling strategy was adopted because the probability density function approaches zero for *ω_D_* > 5 under these loading cases, and its contribution to numerical integration can therefore be neglected. Numerical computation of Equation (24) was subsequently carried out based on the sampled *ω_D_*. The temporal integration step size remained 0.005ms, whereas the integration step for *ω_D_* was set to 5/201, corresponding to an equally‑spaced discretization over the interval [0,5]. For each loading case, 500 such stochastic simulations were conducted, which constitute one round of Monte‑Carlo simulation.

The CAP amplitudes output after Monte‑Carlo simulations for each loading case are illustrated in Fig C. The light‑blue histograms represent the distribution density of CAP amplitudes, and the light‑blue curves denote the KDE fitting of these histograms for a more intuitive visualization of the distribution. Without imposing strong assumptions on the overall distribution of the dataset, the Bootstrap method [1] was adopted to calculate the 95 % confidence interval for the mean of CAP amplitudes, which is depicted as the light‑red shaded region. Specifically, the 500 CAP amplitudes generated from Monte‑Carlo simulations were taken as the original sample. Resampling with replacement was performed to draw 500 observations with the same sample size as the CAP dataset, forming one Bootstrap sample. The mean value of each Bootstrap sample was then computed as the target statistic. The above‑mentioned procedure was independently repeated 10 000 times to obtain 10 000 estimates of the target statistic, which constitute the Bootstrap sampling distribution for the mean CAP amplitude. Finally, these 10 000 estimates were sorted in ascending order, and the 2.5th and 97.5th percentiles were extracted as the lower and upper bounds of the 95 % confidence interval. The original model outputs are plotted as dark‑green solid lines, all lying within the corresponding confidence intervals. This indicates that the electrophysiological responses predicted by our model retain favorable robustness in the presence of fluctuations in damage‑related inputs.

**References**

1.	Efron B. Bootstrap Methods: Another Look at the Jackknife. The Annals of Statistics. 1979;7: 1–26.

Fig A. Results of the sensitivity analysis. (a) Membrane potential curves obtained from five simulation runs with randomly assigned initial conditions *V_0_*, *m_0_*, *h_0_*, and *n_0_*. (b) Magnified view of the region enclosed by the red dashed box in panel (a). (c) Membrane potential curves obtained in individual simulations under a range of values for parameter *C*. (d) Membrane potential curves obtained in individual simulations under a range of values for parameter *k*. The legend indicates the CAP recovery amplitude output in each test, taking the loading case slow‑severe‑30min as an example. Under the baseline parameters and initial conditions specified in the main text, the CAP amplitude for this case is 37.44%.

Fig B The stability of the numerical results derived from the model is demonstrated taking the distribution mean *M* = 1 as an example. (a) presents the results obtained with different *dω_D_* step sizes at a time step of *dt* = 0.005 ms; (b) is an enlarged view of the region enclosed by the red dashed box in subplot (a); (c) shows the results for varying dt step sizes when *dω_D_* = 1/1000; (d) is an enlarged view of the area within the red dashed box in subplot (c).

Fig C Results of the uncertainty analysis of the model using the Monte‑Carlo method. Analyses were performed for all loading cases at the 30 min time point. In each simulation, 200 samples of *ω_D_* were randomly drawn within the numerical integration domain. Integral approximation was then implemented to substitute the infinite integral in Equation (24) of the original manuscript. For each loading case, 500 such stochastic simulations were executed, constituting one round of Monte‑Carlo simulation. The light‑blue histograms in the figure illustrate the distribution density of CAP amplitudes obtained from one round of Monte‑Carlo simulation for each case. The light‑blue curves represent the KDE fitting of the corresponding histograms. The 95 % confidence intervals for the mean values of these CAP amplitudes were estimated via the Bootstrap method and plotted as light‑red shaded regions. The reference results reported in the original manuscript are plotted as dark‑green solid lines, all of which fall within the corresponding 95 % confidence intervals. This observation demonstrates favorable robustness of the model‑predicted electrophysiological responses after injury.
